# Herbarium specimens reveal links between leaf shape of *Capsella bursa‐pastoris* and climate

**DOI:** 10.1002/ajb2.16435

**Published:** 2024-11-06

**Authors:** Asia T. Hightower, Daniel H. Chitwood, Emily B. Josephs

**Affiliations:** ^1^ Department of Plant Biology Michigan State University 612 Wilson Road East Lansing 48824‐1226 MI USA; ^2^ Ecology, Evolution, & Behavior Program Michigan State University 567 Wilson Road East Lansing 48824‐1226 MI USA; ^3^ Plant Resilience Institute Michigan State University East Lansing 48824‐1226 MI USA; ^4^ Department of Horticulture Michigan State University 1066 Bogue Street East Lansing 48824‐1226 MI USA; ^5^ Department of Computational Mathematics, Science, and Engineering Michigan State University 428 S. Shaw Lane East Lansing 48824‐1226 MI USA

**Keywords:** *Capsella bursa‐pastoris*, ecology, evolution, geometric morphometrics, herbaria, leaf shape, precipitation, temperature

## Abstract

**Premise:**

Studies into the evolution and development of leaf shape have connected variation in plant form, function, and fitness. For species with consistent leaf margin features, patterns in leaf architecture are related to both biotic and abiotic factors. However, for species with inconsistent leaf shapes, quantifying variation in leaf shape and the effects of environmental factors on leaf shape has proven challenging.

**Methods:**

To investigate leaf shape variation in a species with inconsistently shaped leaves, we used geometric morphometric modeling and deterministic techniques to analyze approximately 500 digitized specimens of *Capsella bursa‐pastoris* collected throughout the continental United States over 100 years. We generated a morphospace of the leaf shapes and modeled leaf shape as a function of environment and time.

**Results:**

Leaf shape variation of *C. bursa‐pastoris* was strongly associated with temperature over its growing season, with lobing decreasing as temperature increased. While we expected to see changes in variation over time, our results show that the level of leaf shape variation was consistent over the 100 years.

**Conclusions:**

Our findings showed that species with inconsistent leaf shape variation can be quantified using geometric morphometric modeling techniques and that temperature is the main environmental factor influencing leaf shape variation.

It is crucial to understand how complex traits relate to environmental variation, especially in the context of a rapidly changing climate (Anderegg, 2015; Cochrane et al., 2015; Moran et al., 2016; Henn et al., 2018). Leaf shape is a complex trait with variation at the developmental, environmental, and phylogenetic levels (Chitwood et al., 2014; Chitwood and Sinha, 2016; Gupta and Tsiantis, 2018; Lin et al., 2020). For decades, the molecular and morphometric study of leaf shape and its effects on leaf function and plant fitness (Winn, 1999) have been important for advancing crop breeding (Andres et al., 2016; Hao et al., 2022), reducing pesticide use (Rivero‐Lynch et al., 1997; de la Paz Pollicelli et al., 2018), and ultimately improving human health (Key et al., 2008; Broadley and White, 2010). Numerous paleoclimatic and common garden studies have shown that the size and shape of leaves often correlate with temperature and soil moisture on both the local and global scales (Dolph and Dilcher, 1980; Gregory‐Wodzicki, 2000; Huff et al., 2003; Feild et al., 2005; Royer et al., 2008; Gleason et al., 2018; Love and Ferris, 2024). In addition, leaf shape variation is often associated with fitness variation (Bright and Rausher, 2008; Ferris, 2019; Richards et al., 2019).

Leaf shape is a complex trait that is affected by genetic and environmental factors (Blein et al., 2008; Chitwood and Sinha, 2016). Leaf shape is frequently defined by its leaf margin dissections (lobing) (Bilsborough et al., 2011; Peppe et al., 2011). Lobed leaves are simple leaves with leaf margin dissections, making them distinct from compound leaves, which have multiple subunits (leaflets) and discontinuous laminas (Bar and Ori, 2014; Runions et al., 2017). Lobe characteristics are often related to abiotic factors. Generally, increased lobing promotes photosynthesis (Baker and Myhre, 1969; Bhagsari and Brown, 1986; Smith et al., 1997; Kern et al., 2004; Nicotra et al., 2008; Tsukaya, 2018), water transportation (Passioura, 1988; Zwieniecki et al., 2004; Katifori, 2018; Ding et al., 2020; Sakurai and Miklavcic, 2021), and gas exchange (Araus et al., 1986; Pettigrew et al., 1993; Bednarz and van Iersel, 2001; de Boer et al., 2016; Harrison et al., 2020; Tamang et al., 2023; John and Garnica‐Diaz, 2023). Overall, in warm environments, leaves are typically less lobed than leaves in cool environments, while leaves are often smaller in dry environments and larger in wetter environments. (Dolph and Dilcher, 1980; Gregory‐Wodzicki, 2000; Royer et al., 2008, 2009).

Many plant species have leaf shapes that exhibit a traceable and comparable pattern throughout that plant's development and between individual plants (Du et al., 2007). For example, grape vine (*Vitis vinifera*) leaves are palmate and include five major veins (Chitwood et al., 2014), *Arabidopsis thaliana* leaves are simple with either unbroken margins or serrations (Barkoulas et al., 2008, 2017; Koenig and Weigel, 2015; Runions et al., 2017) and cotton (*Gossypium hirsutum*) leaves include four major shapes that differ in carbon fixation depending on other environmental conditions (Pettigrew and Gerik, 2007; de Boer et al., 2016; Andres et al., 2017). However, many species do not have consistent leaf shapes, especially in varying environments, and we know significantly less about the development and evolution of leaf shape in species with inconsistent lobing (Geeta et al., 2012; Kusi and Karsai, 2020). In addition, it is more challenging to study leaf shape in plants with inconsistent lobing; the lack of consistent and/or homologous points on leaves that have variable lobe numbers, lobe depths, and lobe angles makes comparisons among shapes difficult (Valenzuela et al., 2011; Chitwood and Otoni, 2017). Therefore, it is important that we can reliably investigate how leaf shape varies among species with inconsistent lobing across both evolutionary and ecological gradients (Bensmihen et al., 2008). As rising temperatures and increased CO_2_ become more prevalent (Pritchard et al., 1999; Royer, 2012), understanding how species with inconsistent lobing patterns are affected by environmental changes becomes increasingly important.

Geometric morphometrics has been used to summarize shape in terms of a multidimensional landmark configuration, where shapes exist as Cartesian coordinates that can be transformed and compared across two and three dimensions (IIa and Mikeshina, 2002; Adams et al., 2004; Mitteroecker and Gunz, 2009; Jacques and Zhou, 2010; Webster and Sheets, 2010; Polly and Motz, 2016). For many species, the lack of consistency in trait features such as leaf margin lobing or serrations presents challenges in comparing landmarks within and between species because these homologous points may not exist. We addressed this issue with pseudo‐landmarks: points placed between landmarks to estimate curves and to create more continuous representations of shape (Parsons et al., 2009; Budd, 2021).

Herbaria are key resources of trait variation for a wide range and diversity of species over both time and geographic space (Moeller et al., 2007; Moloney et al., 2009; Menne et al., 2012; de Villemereuil et al., 2016; Gutaker et al., 2017; Chen et al., 2018; Heberling et al., 2019; Borges et al., 2020; Sang‐Hun, 2022). Specimens in herbarium collections, which can include whole pressed plants, seeds, fruits, and much more, are a snapshot of the world at the time of collection (Lavoie, 2013; James et al., 2018; Willis et al., 2017; de Villemereuil et al., 2022; Park et al., 2023). A major strength of herbarium specimens is that they provide a view of plant traits from their natural environment, allowing trait changes to be assessed in time and space (Willis et al., 2017; Lang et al., 2019). Through the use of genomic, digitization, and bioinformatics techniques, research with herbarium specimens has increased exponentially (Miller‐Rushing et al., 2004; Besnard et al., 2018; Davis, 2023). Recent work using herbarium specimens has shown that comparisons of the association between traits and the climate across all years, some years, and the climate in the specific year of collection can be used to disentangle genetic and plastic trait changes (Lang et al., 2019; Wu and Colautti, 2022). Here we used leaf shape data from herbarium specimens to quantify and compare leaf shape variation in the weedy allotetraploid *Capsella bursa‐pastoris* (L.) Medik. (Brassicaceae), a species with well‐documented large variations in leaf shape and highly inconsistent leaf margin architecture.

*Capsella bursa‐pastoris* is a model system for investigating within‐species variations in leaf shape across a large environmental range (Figure 1A) (Shull, 1909; Aksoy et al., 1999). It is found in most regions of the world (Neuffer et al., 2018; Choi et al., 2019; Orsucci et al., 2020; Wesse et al., 2021; Cornille et al., 2022) and has incredible variation in leaf shape (Shull, 1909; Neuffer, 1990; Hurka and Neuffer, 1997; Iannetta et al., 2007). Traditionally, leaf shapes in *C. bursa‐pastoris* were identified using a dichotomous leaf key (Lambeth, 1911; Dunwiddie, 1985; Murdock et al., 1988; Enescu, 2017), which showed that leaves from *C. bursa‐pastoris* grown in a common garden could be categorized into shape types (referred to here as the Shull types [Shull, 1909] or the Ianetta types [Iannetta et al., 2007]), and a Mendelian genetic basis was suggested for leaf shape distribution following a temperature and elevation gradient (Neuffer, 1990; Aksoy et al., 1999). However, many studies found leaves that do not fit into one of the four Shull types (Shull, 1909; Aksoy et al., 1999; Begg et al., 2012). In addition, information from common garden experiments alone may miss key morphological information (Moloney et al., 2009; de Villemereuil et al., 2016, 2022), and assigning leaf shapes with dichotomous keys can be subjective, depending on the user (Wiemann et al., 1998; Thyagharajan and Kiruba Raji, 2019; Li et al., 2020). Therefore, a key question we aimed to address is whether leaf shape types can be used to quantitatively assess leaf shape in *C. bursa‐pastoris*. Thus, in this study, we used geometric morphometric techniques to objectively quantify leaf shape based on the two sets of leaf shapes categorized by Shull and by Iannetta (Figure 1B), shape descriptors, climatic factors, and climatic regions (Figure 1A) and investigated leaf shape across the United States over 100 years. For this study, we developed a shape analysis pipeline using pseudo‐landmarks on leaf outlines (Figure 1D) from herbarium specimens of *C. bursa‐pastoris*. We modeled how weather factors affect key leaf shape parameters at different temporal and spatial scales to thoroughly investigate the environmental factors shaping trait distribution.

**Figure 1:**
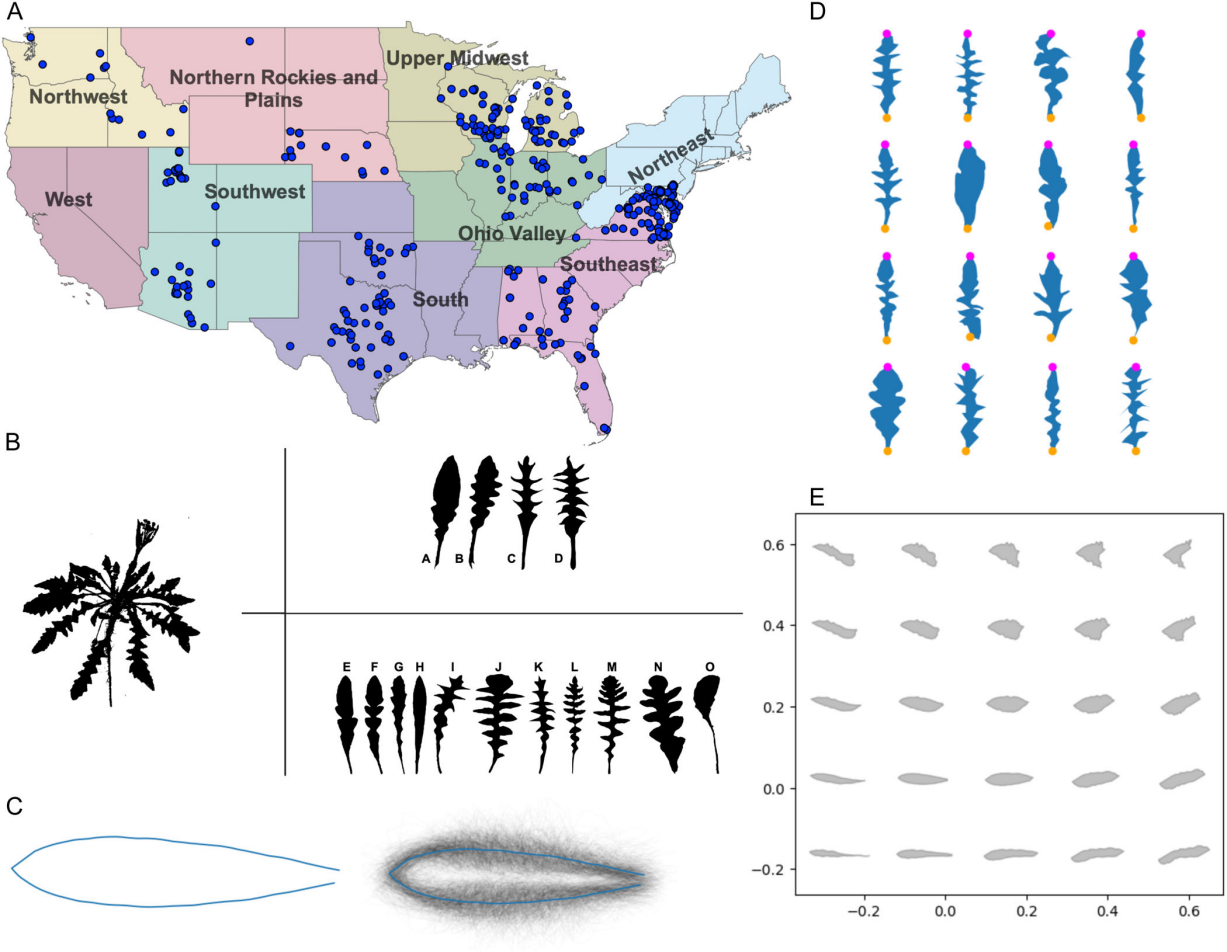
Overview of herbarium specimen selection, leaf shape types, and leaf shape analysis. (A) Map of the continental United States colored by climate region. Blue points represent herbarium specimen collection locations. (B) Schematic of leaf shape types. The left panel includes a representative of the *Capsella bursa‐pastoris* rosette taken from a herbarium specimen. (A–D) Shull leaf shape types Simplex, Rhomboidea, Tenius, and Hetersis. (E–O) Iannetta leaf shape types. (E–H) 1a‐1d, (I–J) 2b‐2b, (K) 3/4, (L) 5, M:6, (N, O) 7a‐7b. (C). Mean leaf shape generated by generalized Procrustes analysis. The left leaf (blue outline) is the overall mean leaf shape; the right leaf is each individual leaf outline overlaid together in black with the mean leaf shape overlaid in blue. (D) Schematic of leaves included in leaf shape analysis, including true landmarks. Outlines of a representative sample of leaves (*N* = 12) included in this study are presented in blue. The two true landmarks, the leaf tip and leaf base, are represented by purple and orange points respectively. (E) Morphospace of theoretical leaves generated by inverse PCA. The morphospace projects five columns and rows of theoretical leaves generated by inverse PCA from leaf outlines included in this study.

## MATERIALS AND METHODS

### Specimen collection and leaf outlines

We examined differences in leaf shape across the continental United States and over 100 years (1921–2021) using 523 digitized herbarium specimens of *C. bursa‐pastoris* (Appendix S1). Each herbarium sample was accessed and downloaded from the online catalog of the Consortium for Midwest Herbaria (2024). We only included samples with legible labels that identified the geographic location of the collection site. To control for differences in rosette development, we only included samples that were flowering when collected. Each state in the continental United States was assessed for sample availability and needed to have at least five potentially usable samples to be included in this study. Our final list of states includes Alabama, Arizona, Delaware, Florida, Georgia, Idaho, Illinois, Indiana, Maryland, Michigan, Montana, Nebraska, Nevada, Ohio, Oklahoma, Oregon, Texas, Utah, Virginia, Washington, and Wisconsin. All NOAA‐defined climatic regions in the United States (Karl and Koss, 1984), except for the West, were represented in this study.

During the second selection step, each specimen was required to include one leaf separated from the whole plant and other leaves, with enough white space to easily outline that leaf. Our final data set included 497 leaves. A condensed list of specimens used, including their climatic regions, is in Appendix S1, and an expanded list of all samples used in this study including the herbarium, label, and weather information can be found in an archived Github repository (DOI: 10.5281/zenodo.13948044). Each leaf was outlined using the segmented line tool in ImageJ (Schindelin et al., 2012). Points were included for both the right and left sides of each leaf, starting at either the right or left end of the petiole, around the leaf, and to the opposite end of the petiole. Each leaf was then saved as an XY coordinate text file. For each leaf, the area, perimeter, length (from tip to visible petiole base), and width were recorded using the ImageJ measurement tool with the settings area, shape descriptors, and perimeter selected.

### Data preparation and generalized Procrustes analysis

To examine differences in leaf shape, we first used the leaf outlines generated by ImageJ in an elliptical Fourier analysis (EFA) using the R package MOMOCS (Bonhomme et al., 2014). However, due to the high degree of variability in leaf lobing and the lack of landmarks, the EFA in MOMOCS was inappropriate for the biological context of our hypothesis. Through the use of harmonic series, we lost lobe definitions between harmonics and failed to generate a usable morphospace. Instead, we created a leaf shape pipeline to address the variability in leaf lobing and the lack of landmarks.

Alternatively, we could have measured leaf shape using convex hull analysis. In convex hull analysis, a convex hull is generated by connecting points on the leaf outlines (Ferris et al., 2015). From a geometric morphometric perspective, the points defining the convex hull are already included in our outlined data set, so the convex hill method does not add extra information.

We analyzed the coordinate file of each outlined leaf shape with a shape analysis pipeline using equidistant points in Python (Perez and Granger, 2007) using Jupyter Notebook (Kluyver et al., 2016). This pipeline included importing leaf outline as coordinate text files, interpolating all points, and performing generalized Procrustes analysis (GPA) (Gower, 1975; Dijksterhuis and Gower 1991; Igual et al., 2014). To perform the landmark analysis, we first needed to orient each leaf so that each leaf was rotated and facing the same direction. To do this, we found the indices (coordinate values/points) that represented the tip and base of each leaf. These indices were then re‐indexed so that each leaf began at the base. Each leaf was rotated so that all leaf tips and leaf bases were facing the same direction. Due to the variability in leaf shapes, we could only include two true landmarks for landmark analysis: the tip and the base of each leaf. Therefore, we assigned 100 equidistant pseudo‐landmarks from leaf tip to leaf base (left side of leaf) and then from leaf base to leaf tip (right side of leaf) so that each leaf included the same number of points. These equidistant points allowed us to compare points on each side of the leaf along a proximal distal axis. We then performed a GPA on these re‐indexed shapes. During the GPA, each leaf was scaled and transformed to be compared to an arbitrary starting leaf (the first leaf in our data set). After transformation, we calculated the Procrustes distance and generated a “mean” leaf shape, which is the average of all leaf shapes included in this data set. This process iterates across all leaves in our data set until a Procrustes threshold is reached. The final products of the GPA include a final Procrustes distance and a new set of Cartesian coordinates based on the scaled and transformed leaves. From the GPA, we produced a mean leaf shape for the continental United States (Figure 1C). We defined archetypal leaves representing the four Shull leaf shape types (Shull, 1909) and the seven Iannetta et.al. shape types (Iannetta et al., 2007). We then used a GPA to match each leaf in our study to an archetypal leaf from both type categories. The final products of this pipeline were a series of CSV files that included “best matches” for each of the type categories based on the Procrustes distance between the herbarium leaf and the archetypal leaf, circularity values (defined below), and aspect ratio values.

### Principal component analysis and shape descriptors

After the GPA, we performed principal component analysis (PCA) on the re‐indexed leaves. We then performed inverse PCA to plot theoretical (eigen) leaves. Using the inverse PCA theoretical leaves, we defined a morphospace function to plot theoretical leaves from PC1 and PC2 eigenvalues along the PC space (Figure 1E). We measured shape descriptors to describe differences in lobing and size between each leaf. We used circularity (Circ), calculated as Circ = (4*π* × Area) ÷ Perimeter^2^ to measure lobing between leaves. In this equation, a value of 1 describes a perfect circle, and values below 1 indicate increased lobing. We also used aspect ratio (AR) to measure changes in size (AR = Width ÷ Length) for each leaf. Lower aspect ratio values suggest a leaf is wider and shorter; higher aspect ratio values suggest a leaf is longer and narrower.

### Weather data collection

We collected the average temperature (AT), maximum temperature (MAX), minimum temperature (MIN), and average precipitation accumulation (AP) for the location of each plant sample for each time range. We included three time‐range models in which we collected weather data. (1) The date of collection (DOC) model included each weather factor as it was reported on the date of collection for each specimen. (2) The growing season (GS) model included the average of the daily measurements of each weather factor, collected over 6 months, beginning 6 months before the date of collection and ending on the date of collection. (3) The year‐long (YL) model included the average of the daily measurements of each weather factor, excluding the average temperature for the year (AT), beginning 1 year (365 days) before the date of collection and ending on the date of collection.

To collect weather data, we generated a list of coordinates (latitude and longitude) for all specimens. We used the R package rnoaa (Sparks et al., 2017; Chamberlain and Hocking, 2024) to download daily station data from the NOAA ghcnd database (Peterson et al., 1998). We then found up to 200 stations within a 50‐mile radius of each location. We then separated each set of stations by city and found all station ID information for each city. Using the filtered station IDs, we collected daily average temperature (AT), maximum temperature (MAX), minimum temperature (MIN), and daily average precipitation (AP) accumulation from 1 January 1920 to 1 January 2021 for each city. We then calculated the average weather measurements across all stations for each city. Temperature was reported by NOAA in 1/10th degrees, so we multiplied these values by 10. Average precipitation (AP) was reported to the 10th of an inch. We converted AP to 1 inch and log‐transformed the data. To find both the GS and YL weather data points, we used the same process as above to pare our original comprehensive data set. To determine the YL weather, we calculated the mean as (Average temperature of hottest month + Average temperature of coldest month)/2. We used the R package zoo (Zeileis and Grothendieck, 2005) to find the beginning date of the previous 6 months or previous year. To determine the weather on the date of collection (DOC), we used the same process as above, by searching for only the weather data reported in each city on the date of collection for each specimen.

### Statistical analyses

All analyses were performed with R version 4.2.3 (RStudio Team, 2020; R Core Team, 2021). We used Pearson's *χ* ^2^ test of association to determine the strength of association between each leaf shape type category (Shull and Iannetta) and with climate region using the frequency of leaf shape type in each climate region. We also conducted one‐sided *t*‐tests and ANOVAs to determine associations between climate region and leaf shape using the shape descriptor circularity or aspect ratio as a proxy for leaf shape.

To estimate differences in leaf shape by shape descriptors, we performed an ANOVA on each weather × time model. These models included:

$$\begin{array}{rll} \mathrm{GS} & = & \mathrm{Shape}\;\mathrm{Descriptor}\sim {\mathrm{AT}}_{\mathrm{GS}}+{\mathrm{MAX}}_{\mathrm{GS}}+{\mathrm{MIN}}_{\mathrm{GS}}\\ & & +{\mathrm{AP}}_{\mathrm{GS}}+\mathrm{Climate}\;\mathrm{Region}\\ \mathrm{YL} & = & \mathrm{Shape}\;\mathrm{Descriptor}\sim {\mathrm{AT}}_{\mathrm{YL}}+{\mathrm{MAX}}_{\mathrm{YL}}+{\mathrm{MIN}}_{\mathrm{YL}}\\ & & +{\mathrm{AP}}_{\mathrm{YL}}+\mathrm{Climate}\;\mathrm{Region}\\ \mathrm{DOC} & = & \mathrm{Shape}\;\mathrm{Descriptor}\sim {\mathrm{AT}}_{\mathrm{DOC}}+{\mathrm{MAX}}_{\mathrm{DOC}}\\ & & +{\mathrm{MIN}}_{\mathrm{DOC}}+{\mathrm{AP}}_{\mathrm{DOC}}+\mathrm{Climate}\;\mathrm{Region}\\ {\mathrm{IN}}_{\mathrm{GS}} & = & \mathrm{Shape}\;\mathrm{Descriptor}\sim \mathrm{GS}\;\mathrm{model}+{\mathrm{AT}}_{\mathrm{GS}}\times {\mathrm{AP}}_{\mathrm{GS}}\\ {\mathrm{IN}}_{\mathrm{YL}} & = & \mathrm{Shape}\;\mathrm{Descriptor}\sim \mathrm{YL}\;\mathrm{model}+{\mathrm{AT}}_{\mathrm{YL}}\times {\mathrm{AP}}_{\mathrm{YL}}\\ {\mathrm{IN}}_{\mathrm{DOC}} & = & \mathrm{Shape}\;\mathrm{Descriptor}\sim \mathrm{DOC}\;\mathrm{model}\\ & & +{\mathrm{AT}}_{\mathrm{DOC}}\times {\mathrm{AP}}_{\mathrm{DOC}}, \end{array}$$

where IN models include the interaction between the average temperature and average precipitation for each time‐range. A parametric variance test, Tukey's honestly significant difference (HSD) test, was performed to determine differences in shape descriptors between climate regions. We then performed delta Akaike information criterion (AIC) model comparison (Mazerolle, 2023) to find the best model for explaining differences in variance between shape descriptors. For the GS, YL, and DOC models described above, we included the same model without climate region in the AIC model comparison. We performed one‐way ANOVA on shape descriptors to determine their respective associations with Climate Region using the following equations: Climate × Circ = Circ ∼ Climate Region and Climate × AR = AR ∼ Climate region. We then performed a one‐sided *t*‐test on mean circularity and mean aspect ratio (AR) for each climate region.

## RESULTS

### *Capsella bursa‐pastoris* leaf shapes vary continuously

To confirm whether leaves fall into distinct shapes as previously found or have continuous patterns of variation, we analyzed the outline of one leaf from 497 specimens of *C. bursa‐pastoris* from herbaria across the continental United States (Figure 1D) using our shape analysis pipeline. Due to their high degree of intraspecific shape variation, the leaves did not have any discernible leaf margin architectural features in the “mean” leaf (average of all leaf shapes) generated by the generalized Procrustes analysis (GPA; Procrustes distance) (Figure 1C). This lack of discernible features was also apparent in the “eigen” leaf (theoretical leaf) representations of the morphospace (Figure 1E). The lack of definition in the theoretical leaves is due to the high degree of lobe number and architecture variability (Figure 1C) coupled with the small sample size. Therefore, we did not consider the overall shape further in this study. However, even though lobes were not represented in morphospace representations of the leaves, a pseudo‐landmark approach still comprehensively measured the outline of the leaf (for example, see Chitwood et al., 2014). Using our leaf shape analysis pipeline, we measured the traditional morphometric traits of leaf length, width, area, aspect ratio, and circularity, and using pseudo‐landmarks, we generated Procrustes distances between all leaves to determine shape differences among all the leaves. We used GPA to comprehensively compare each herbarium leaf outline to archetypal Shull (Figure 1B[A–D]) or Iannetta types (Figure 1B[E–O]), assigning leaves to categories based on the smallest Procrustes distance to an archetypal leaf. Almost all leaves (94%, *N* = 470) matched the “Rhomboidea” Shull type best, and 78% (*N* = 388) of leaves best matched the “Type 3” Iannetta type, consistent with previous common garden experiments that found that these were the most common leaf shape types (Shull, 1909; Neuffer, 1990; Hurka and Neuffer, 1997; Iannetta et al., 2007; Neuffer et al., 2018). Additionally, we measured leaf shape (lobing) using circularity (Circ) and leaf size using aspect ratio (AR).

**Figure 2:**
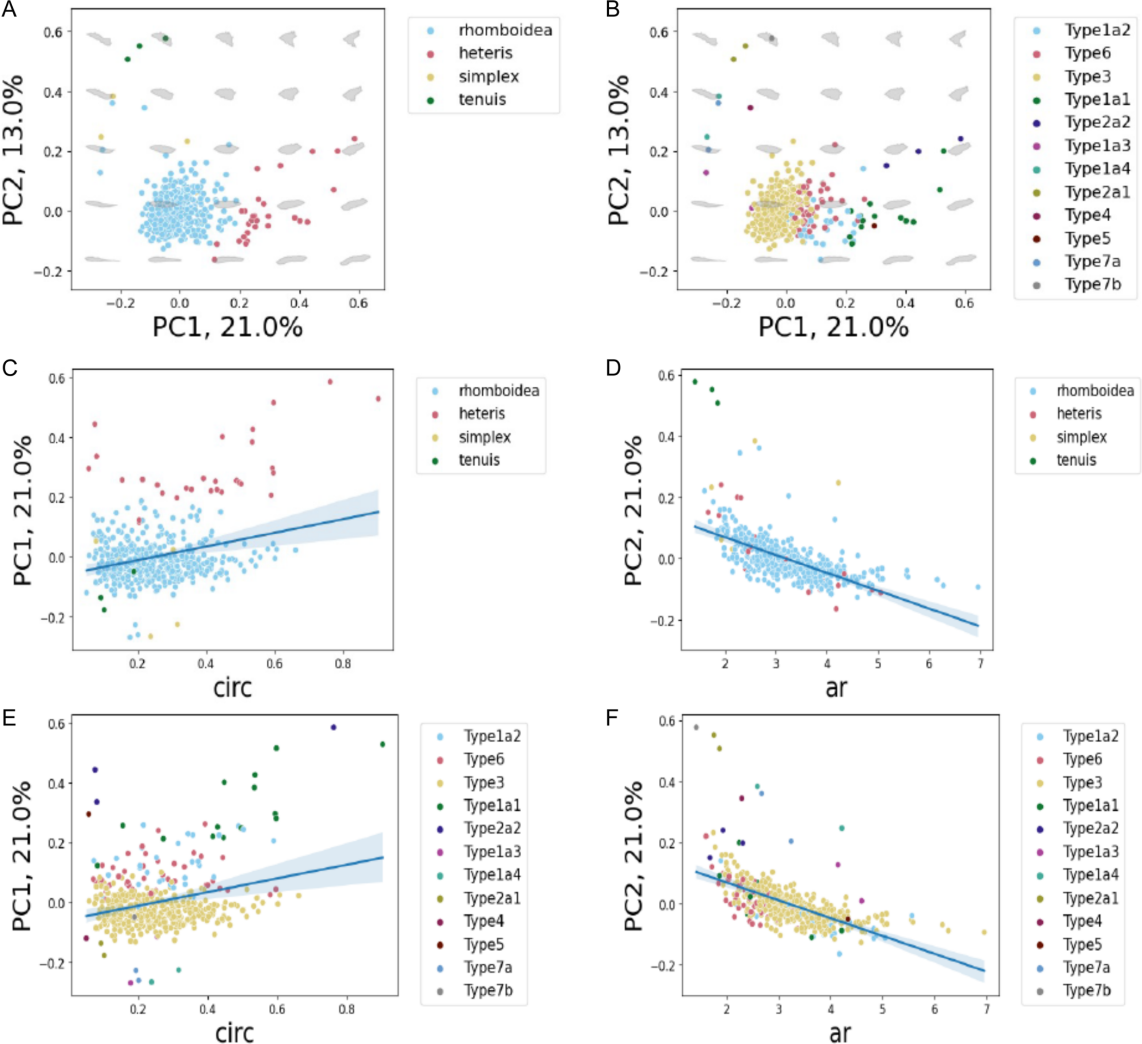
*Capsella bursa‐pastoris* leaf morphospace, leaf shape types, circularity, and aspect ratio. (A) Morphospace PCA of leaves as classified by Shull leaf shape types. (B) Morphospace PCA of leaves as classified by Iannetta leaf shape types. (C, E) Graph of circularity (circ) against PC1. Leaves are colored by their respective leaf shape type categories: Shull types (C) and Iannetta types (E). (D, F) Graph of aspect ratio (ar) by PC2, leaves colored by their respective leaf shape type categories: Shull types (D) and Iannetta types (F). The blue line represents the fitted linear regression; the gray band represents the 95% confidence interval.

The morphospace PCA generated with the theoretical leaves from the GPA but not the aspect ratio or circularity measurements revealed that leaf shapes varied continuously, and there was considerable overlap in leaf shape (Figure 2A, B). PC1 and PC2 explained 21% and 13% of the variance in shape, respectively. Both the “Rhomboidea” and “Type 3” shape categories spanned a majority of the available PC space suggesting that focusing on shape types will miss a lot of within‐type leaf shape variation (Figure 2A, B). In addition, the “Rhomboidea” type encompassed the entire range of available shape descriptors (circularity and aspect ratio) values in this study (0.05758 to 0.76106 circularity values and 1.712 to 6.956 aspect ratio values). Pearson's *χ* ^2^ test of association revealed that only the Shull leaf shape types were correlated with climate region (Cramer's *V* = 0.343, *P* = 2.03 × 10^−17^). This pattern of continuous variation, along with evidence that major shape types were found in every climate region and consistently across time, suggests that type is not the most effective way to investigate how the environment relates to leaf shape.

**Figure 3:**
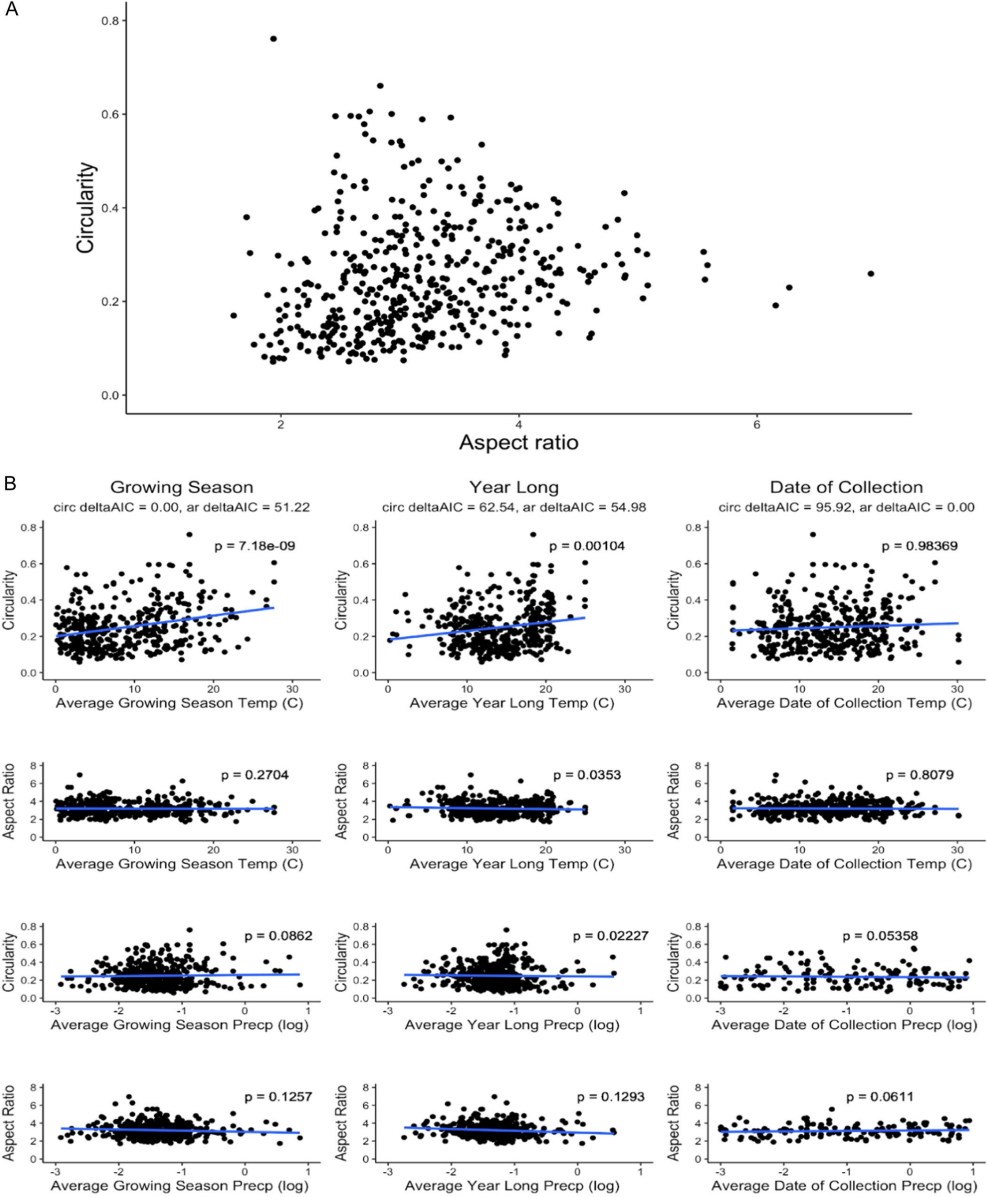
Modeling circularity and aspect ratio for *Capsella bursa‐pastoris* leaves. (A) Circularity and aspect ratio modeled together show the boundaries of shape and size through the triangular shape of the data. Leaves can be short and wide (at the left tail of the data) and long and narrow (at the right tail of the data). (B) Effects of temperature and precipitation on circularity and aspect ratio. The blue line represents the linear regression. The first column includes circularity and aspect ratio by the growing season (GS) temperature and precipitation. The second column includes circularity and aspect ratio by the year‐long (YL) temperature and precipitation. The third column includes circularity and aspect ratio by the date of collection (DOC) temperature and precipitation. The model comparison deltaAIC is included for each weather factor × time model for both shape descriptors. The best model for explaining variance in circularity (lobing) was the GS model that includes climate region, with a deltaAIC score of 0. The best model for explaining variance in aspect ratio (size) was the DOC model including climate region.

The theoretical leaves of the morphospace PCA were separated continuously along PC1 and significantly associated with circularity (*P* = 9.14 × 10^−12^, Figure 2C, E; Appendix S2). The theoretical leaves also were separated continuously along PC2 and significantly associated with aspect ratio (*P* = 2 × 10^−16^, Figure 2D, F; Appendix S2). Circularity and aspect ratio were also moderately positively correlated with each other (Spearman's *ρ* = 0.302, *P* = 5.691 × 10^−1.2^). There was strong constraint in change in circularity at extreme values of aspect ratio and more variation in circularity at intermediate values of aspect ratio (Figure 3A). This pattern suggests that leaves can reach a maximum width (at low AR values) and a maximum length (at high AR values) only in highly lobed leaves, consistent with potential biological constraints for *C. bursa‐pastoris* leaf shape.

Overall, the results of the geometric morphometric analysis suggest that both the Shull and Iannetta leaf shape types are less morphologically distinct than previously thought. Therefore, descriptive categorizations of leaf types are not meaningful for shape comparisons and will not be used going further in this study. Instead, we will focus on circularity and aspect ratio since they better described the range of leaf shape variation throughout the PC space.

### Leaf shapes varied by climate region and growing season temperature

To further investigate the relationship between leaf shape descriptors and climate region, we performed one‐sided *t*‐tests to determine whether mean circularity and mean aspect ratio were individually significantly different among climate regions. Leaves from different climate regions had significantly different mean circularity (*P* = 3.097 × 10^−8^) and mean aspect ratio (*P* = 2.294 × 10^−10^). In the one‐way ANOVA and post hoc tests to determine which regions were had leaves with significantly different circularity and aspect ratios (Appendix S3), circularity was significantly different between the South and Northeast (*P* = 1.4 × 10^−6^), South and Southeast (*P* = 1.29 × 10^−5^), and South and Upper Midwest (*P* = 0.007.6508 × 10^−3^). Aspect ratio was significantly different between the Upper Midwest and Northeast (*P* = 4.4644 × 10^−3^). Overall, these results suggest that leaf shape differs among regions, which led us to investigate the environmental factors that contributed to this variation.

To test which environmental factors best explained phenotypic variation in leaf shape, we modeled shape descriptors as a function of average temperature (AT), maximum temperature (MAX), minimum temperature (MIN), and average precipitation (AP). We investigated temperature and precipitation at three time scales: the environmental conditions of the 6 months preceding collection (growing season, or GS), the environmental conditions of the year before collection (year long [YL]), and environmental conditions on the date of collection (DOC). We compared the growing season and year‐long models because previous work has shown that the environmental conditions of the specific time of year in which *C. bursa‐pastoris* grows is more useful for determining the ecological niche than year‐long data (Wilson Brown and Josephs, 2023). The DOC model acts as a negative control because we did not expect the temperature or precipitation on the date of collection to affect leaf shape variation.

We used AIC model selection to determine which model best explained the variance in circularity and aspect ratio across the continental United States (Figure 3B). The best‐fit model for explaining variance in circularity included every parameter in the GS model with no interaction effects. In this model, circularity increased as the average temperature (*P* = 7.15 × 10^−10^) and maximum temperature increased (*P* = 5.38 × 10^−12^). The second‐best model was the YL model that included every parameter with no interaction effects (*P* = 0.00153).

The DOC and interaction models showed no significant differences in circularity across any of the included parameters. For aspect ratio, the DOC model was the best‐fit model and included every parameter. However, there were no significant associations between any of the temperature or precipitation variables and aspect ratio in the DOC model. For all models, precipitation was not significantly associated with either circularity or aspect ratio. Therefore, precipitation was not predictive of shape or size in our data set.

### Growing season temperature explains leaf shape variation throughout the continental U.S. and by region

**Figure 4:**
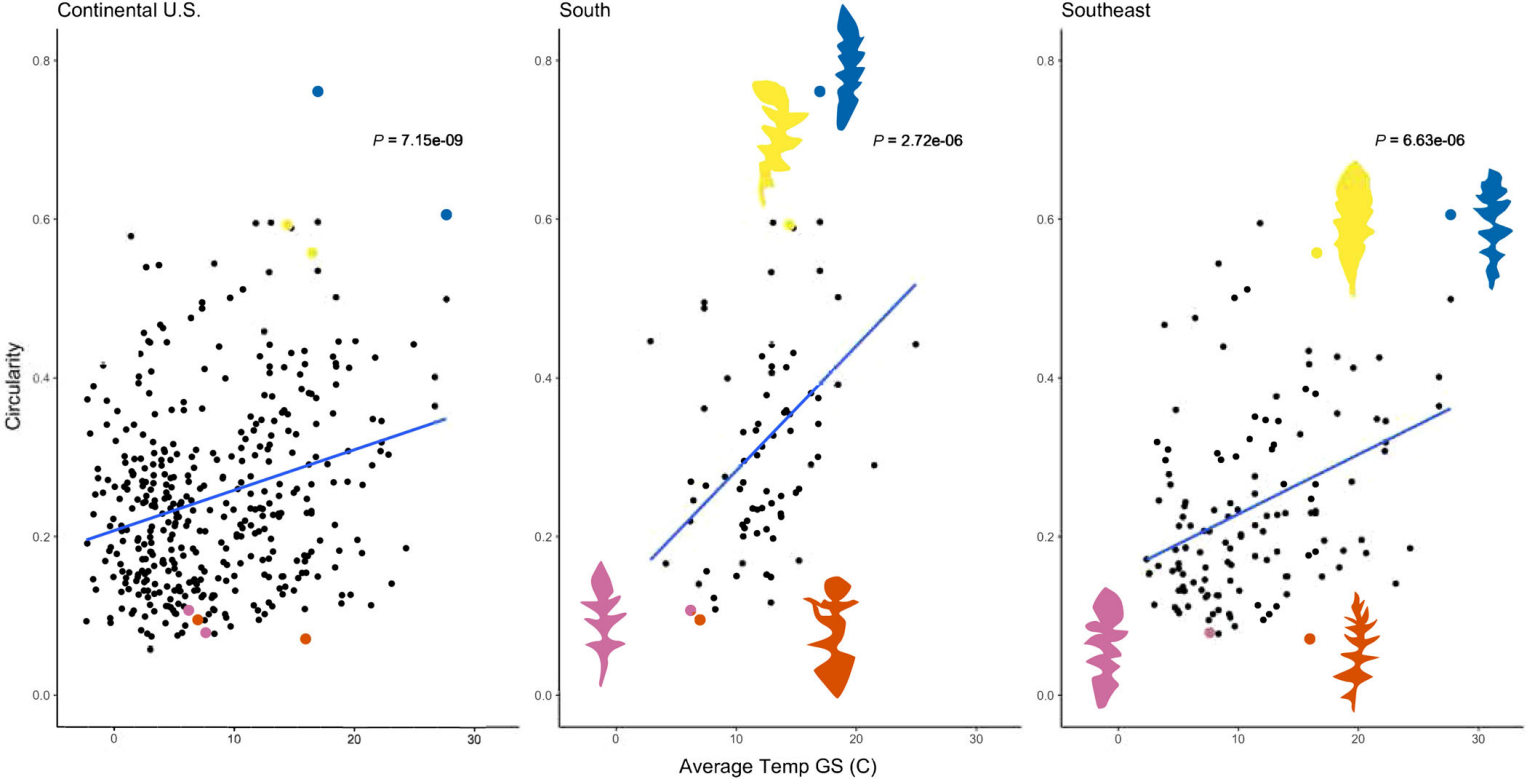
The relationship between average growing season (GS) temperature and circularity (circ) across all samples (left), in the South (middle) and in the Southeast (right). In all panels, the blue line represents the fitted linear regression. The two highest and two lowest circularity values for the South and Southeast regions are colored in all three panels and represented by leaf images. Blue = highest circ, yellow = second highest circ, pink = second lowest circ, orange = lowest circ.

Model selection revealed that the temperature in the 6 months before collection (GS) explained the variation in leaf shape better than the year‐long temperature (YL). However, the relationship between GS temperature and leaf shape was not consistent across the continental United States. The South and Southeast climatic regions had the strongest associations between circularity (lobing) and average temperature (Figure 4A; Appendix S4). In the additional six climatic regions, there was weak to no correlation between circularity and temperature. The largest range of circularity values was seen in the South (0.0951–0.7611) and Southeast regions (0.0711–0.6057).

The large range in circularity and strong association between temperature and shape could be due to a larger sample size in the Southeast but not the South. The Southeast included 152 individuals, the South 78 individuals, the Upper Midwest 82 individuals, Ohio Valley 57 individuals, Northeast 51 individuals, Southwest 40 individuals, Northern Rockies and Plains 20 individuals, and the Northwest included 16 individuals. A summary of individuals by climate region is included in Appendix S5.

### Leaf shape variation remained consistent over the 100 years

Leaf shape did not change over time across the continental United States although there were some changes within some climatic regions. Circularity increased over time in the South (*P* = 1.08 × 10^−8^) and Southwest (*P* = 0.00683) regions and decreased over time in the Northwest (*P* = 0.00628), Northern Rockies and Plains (*P* = 0.02929), Upper Midwest (*P* = 0.02093), and Southeast (*P* = 0.03362). Aspect ratio values followed a similar trend; they did not change over time across the continental United States, but increased in the Upper Midwest (*P* = 6.69 × 10^−05^) and Northwest (*P* = 0.0225). Overall, leaf shape variation was sustained at the continental scale for the 100‐year period (Appendix S6).

## DISCUSSION

In this study, we found tremendous leaf shape variation within *C. bursa‐pastoris* using tools that allowed us to systematically measure shape variation in scanned herbarium samples. We showed that this variation is not well described by previous classification systems and instead propose that quantitative measures of lobing are the best way to quantify shape in this species. We linked this leaf shape variation (circularity) to temperature and showed that this relationship, while significant across the North American range, is driven by associations within specific regions. The AIC model selection ranking showed that the GS model had the greatest effect on circularity by temperature factors, while the DOC model had the greatest effect on aspect ratio; however, none of the terms in the model were significant. Additionally, the DOC model, which was included as a negative control, showed only a small but statistically significant effect on circularity and not aspect ratio, where only precipitation was marginally significant (*P* = 0.053). These results suggest that there is no significant association between temperature and precipitation on aspect ratio in our study and that the effects of precipitation on circularity are inconclusive. While shape varied in space, we did not see significant changes in shape or the extent of variation in shape across time. Our results have clear implications for identifying the environmental factors contributing to intraspecific variation and providing a guide for systematically investigating shape variation in species with variable leaf shapes.

Historically, categories of leaf shape types have been used to subjectively categorize leaves (Shull, 1909; Iannetta et al., 2007; Shi et al., 2019; Zhang et al., 2019; Schrader et al., 2021). The *C. bursa‐pastoris* studies (Shull, 1909; Iannetta et al., 2007) had an overrepresentation of one leaf shape type and intermediate types that did not fit into one or more categories. Our Procrustes distance‐based results suggest that there is substantial shape variation within categories. Within the Rhomboidea type alone, there is the full range of circularity found in this study. Therefore, distinctions made by category types may not be as meaningful as distinctions made by quantitative factors such as circularity, where different shape types may be represented by one circularity value (Felsenstein, 1973; Quinteros et al., 2006; Parins‐Fukuchi, 2018). However, differences in leaf shape types may become more pronounced with the addition of more samples.

Instead of using shape categories, we used a pseudo‐landmark approach to investigate leaf shape. Traditional landmark analysis of complex leaf shapes like those of *C. bursa‐pastoris* can be difficult because there are inconsistencies in trait features such as lobing depth, lobe/leaflet number, and lobe/leaflet size that make it challenging to assign landmarks. The use of pseudo‐landmarks allows for comparisons between landmark points regardless of any inconsistencies in shape (Lawing and Polly, 2010; Dujardin et al., 2014). These approaches will be broadly useful because other plant species also have inconsistent leaf shapes. For example, *Arabidopsis lyrata* has varying leaf lobes and serrations (Vergeer and Kunin, 2011), and *Cardamine hirsuta* has varying leaf shape and leaflet number (Canales et al., 2010). Additionally, leaf shape analyses that utilize outlines can be paired with morphometric techniques such as EFA/MOMOCS (Bonhomme et al., 2014) and convex hull comparisons (Ferris et al., 2015) to better distinguish between groups, particularly for more‐lobed versus less‐lobed. These techniques are often paired with topological data analysis of leaf shape as done by Li et al. (2018).

While herbaria provide a remarkable source of plant traits and other data, there are some limitations to the conclusions that can be made from this data. The current 497 samples included in this study are biased in their collection times and locations. Most samples were collected within and around more urban areas, and the majority of repeated collection sites and collection times resulted from class projects at universities (Appendix S7). This bias has been well documented in herbarium studies (Moerman and Estabrook, 2006; Loiselle et al., 2008; Daru et al., 2018; Panchen et al., 2019; Williams and Pearson, 2019; Meineke and Daru, 2021) and highlights the need for repeated and sustained collections over an expanded collection range. In addition, traits measured from herbarium samples will be affected by the genotype and the environment of the individual, making it difficult to distinguish the underlying source of trait variation. Future work using common gardens, like that of Gupta et al. (2020), will be key for understanding how environment shapes leaf shape variation in *C. bursa‐pastoris*.

As one of the most invasive plant species in the world, *C. bursa‐pastoris* colonized, established, and flourished in a wide range of habitats and climates (Cornille et al., 2016, 2022; Wesse et al., 2021; Wilson Brown and Josephs, 2023). Some researchers have suggested that high plasticity may help *C. bursa‐pastoris* persist across a wide range of environments (Choi et al., 2019; Cornille et al., 2022) For example, Choi et al. (2019) observed strong phenotypic plasticity for specific leaf area and leaf length in response to temperature and soil moisture in *C. bursa‐pastoris* and found evidence of selection for plasticity for specific leaf area. In addition, there is evidence that leaf type and traits such as thickness and stomatal density vary genetically across the *C. bursa‐pastoris* range (Neuffer et al., 2018). Here, we contribute to these previous results by showing that shape can be best described quantitatively and that leaf circularity correlates with temperature and differs between climatic regions. While associations between leaf shape and temperature suggest that shape is related to fitness in different types of environments, future work to determine any direct links of leaf shapes to fitness is needed to comprehensively understand the ecological importance of this trait during invasion.

The variations in leaf shape also suggest that a genetic mechanism underlies leaf shape response to the environment, although we did not measure genetic sequence variation directly in this study. Previous research on the genetic basis for Shull leaf shape types suggests that two Mendelian loci with two alleles each control the elongation of primary lobes (allele A) and the division of lobes (allele B) (Neuffer, 1990; Neuffer and Meyer‐Walf, 1996). However, here we found continuous variation in leaf shape, which would suggest the genetic mechanism of patterning leaf margins is not Mendelian or that it is strongly affected by environmental factors that varied across samples. Recent studies into the genetics of leaf lobing in *Cardamine hirsuta*, *Capsella grandiflora*, *Capsella rubella*, and other members of the lineage I Brassicaceae family has revealed the importance of *REDUCED COMPLEXITY 1* (*RCO*) (Sicard et al., 2014; Streubel et al., 2018). For *Capsella* species, the *RCO‐A* gene induces the formation of lobes and reduces the blade surface area (Sicard et al., 2014; Streubel et al., 2018). In *C. grandiflora* specifically, *RCO‐A* expression increases dramatically in low temperatures, almost 10 times the normal expression at 20 C (Sicard et al., 2014; Streubel et al., 2018). The *RCO‐B* gene induces the formation of serrations and is involved in proximal–distal leaf patterning in *C. grandiflora* and *C. rubella* (Sicard et al., 2014; Streubel et al., 2018). The presence of these patterns of *RCO* expression in *C. bursa‐pastoris* would be strong evidence for leaf shape variation on a gene‐by‐environment basis. Currently, *RCO* has yet to be characterized both genetically and functionally in *C. bursa‐pastoris*.

Ultimately, fully characterizing how genetic variation and environmental variation contribute to leaf shape variation will be key for understanding the maintenance of variation for leaf shape across the North American range. While leaf shape variation is common, it has been challenging to link this variation to specific agents of selection (Campitelli and Stinchcombe, 2013). The few studies that have found variation in selection on leaf shape across habitats were done on relatively small spatial scales (Ferris and Willis, 2018; Richards et al., 2019), but Walter et al. (2023) provided an example where selection on leaf shape was in the same direction across elevations. These studies, along with our finding that the relationship between temperature and leaf shape varies among regions, suggest that while leaf shape may often be important for fitness, the selective forces acting on shape may vary among regions. This heterogeneity across large landscapes will contribute to the challenge of understanding the forces contributing to variations in shape. This work and other basic studies like it are necessary first steps to understanding the biological mechanisms and potential consequences of climatic changes on leaf form and function and potentially on plant fitness.

## CONCLUSIONS

Our work revealed a continuum of leaf shapes for *C. bursa‐pastoris* and that discrete leaf shape types are more arbitrary than previously thought. Leaf shape was correlated with the growing season temperature of the plant, although this relationship varied among geographic regions, suggesting that temperature has a large effect on leaf shape variation. Additionally, while our results did not show changes in leaf shape over time, we did see that the leaf shape variation persisted over the 100 years included in this study. Finally, the use of herbarium samples and the leaf shape analysis pipeline created for this study allowed us to compare complex, variable leaf shapes in an easy and less computationally intense way. This shape analysis pipeline will allow for further studies of complex shapes that were previously too difficult to pursue.

## AUTHOR CONTRIBUTIONS

A.T.H., D.H.C., and E.B.J. designed the research. A.T.H. performed the research, data collection, and analysis and wrote the manuscript with advice from D.H.C. and E.B.J.

## CONFLICTS OF INTEREST STATEMENT

The authors declare they have no competing financial interests.

## Supporting information


**Appendix S1.** Herbarium information for all *Capsella bursa‐pastoris* specimens included in this study.

**Appendix S2.** Circularity and aspect ratio cluster within the morphospace PCA.

**Appendix S3.** Circularity and aspect ratio values are significantly different between climatic regions.

**Appendix S4.** Circularity is strongly associated with average growing season temperature and by climate region.

**Appendix S5.** Sample sizes of individuals collected from each climate region.

**Appendix S6.** Circularity and aspect ratio varied over the 100 years studied.

**Appendix S7.** Individuals collected on or near a university campus.

## Data Availability

The data that support the findings of this study along with all code to do the analysis are openly available in a GitHub repository: DOI: 10.5281/zenodo.13948043.

## References

[ajb216435-bib-0001] Adams, D. C. , J. F. Rohlf , and D. E. Slice . 2004. Geometric morphometrics: ten years of progress following the ‘revolution’. Italian Journal of Zoology 71: 5‐16.

[ajb216435-bib-0002] Aksoy, A. , W. H. G. Hale , and J. M. Dixon . 1999. Towards a simplified taxonomy of *Capsella bursa‐pastoris* (L.) Medik. (Brassicaceae). Watsonia 22: 243‐250.

[ajb216435-bib-0003] Anderegg, W. R. L. 2015. Spatial and temporal variation in plant hydraulic traits and their relevance for climate change impacts on vegetation. New Phytologist 205: 1008‐1014. 25729797 10.1111/nph.12907

[ajb216435-bib-0004] Andres, R. J. , D. T. Bowman , D. C. Jones , and V. Kuraparthy . 2016. Major leaf shapes of cotton: genetics and agronomic effects in crop production. Journal of Cotton Science 20: 330‐340.

[ajb216435-bib-0005] Andres, R. J. , V. Coneva , M. H. Frank , J. R. Tuttle , L. F. Samayoa , S.‐W. Han , B. Kaur , et al. 2017. Modifications to a *LATE MERISTEM IDENTITY1* gene are responsible for the major leaf shapes of Upland cotton (*Gossypium hirsutum* L.). Proceedings of the National Academy of Sciences, USA 114: E57‐E66. 10.1073/pnas.1613593114 PMC5224360 27999177

[ajb216435-bib-0006] Araus, J. L. , L. Alegre , L. Tapia , and R. Calafell . 1986. Relationship between leaf structure and gas exchange in wheat leaves at different insertion levels. Journal of Experimental Botany 37: 1323‐1333.

[ajb216435-bib-0007] Baker, D. N. , and D. L. Myhre . 1969. Effects of leaf shape and boundary layer thickness on photosynthesis in cotton (*Gossypium hirsutum*). Physiologia Plantarum 22: 1043‐1049.

[ajb216435-bib-0008] Bar, M. , and N. Ori . 2014. Leaf development and morphogenesis. Development 141: 4219‐4230. 25371359 10.1242/dev.106195

[ajb216435-bib-0009] Barkoulas, M. , A. Hay , E. Kougioumoutzi , and M. Tsiantis . 2008. A developmental framework for dissected leaf formation in the *Arabidopsis* relative *Cardamine hirsuta* . Nature Genetics 40: 1136‐1141. 19165928 10.1038/ng.189

[ajb216435-bib-0010] Bednarz, C. W. , and M. W. v. Iersel . 2001. Temperature response of whole‐plant CO_2_ exchange rates of four upland cotton cultivars differing in leaf shape and leaf pubescence. Communications in Soil Science and Plant Analysis 32: 2485‐2501.

[ajb216435-bib-0011] Begg, G. S. , J. Wishart , M. W. Young , G. R. Squire , and P. P. M. Iannetta . 2012. Genetic structure among arable populations of *Capsella bursa‐pastoris* is linked to functional traits and in‐field conditions. Ecography 35: 446‐457.

[ajb216435-bib-0013] Bensmihen, S. , A. I. Hanna , N. B. Langlade , J. L. Micol , A. Bangham , and E. S. Coen . 2008. Mutational spaces for leaf shape and size. HFSP Journal 2: 110‐120. 19404477 10.2976/1.2836738 PMC2645570

[ajb216435-bib-0015] Besnard, G. , M. Gaudeul , L. Sébastien , S. Muller , G. Rouhan , A. P. Sukhoruko , A. Vanderpoorten , and F. Jabbour . 2018. Herbarium‐based science in the twenty‐first century. Botany Letters 165: 323‐327.

[ajb216435-bib-0016] Bhagsari, A. S. , and R. H. Brown . 1986. Leaf photosynthesis and its correlation with leaf area 1. Crop Science 26: 127‐132.

[ajb216435-bib-0017] Bilsborough, G. D. , A. Runions , M. Barkoulas , H. W. Jenkins , A. Hasson , C. Galinha , P. Laufs , et al. 2011. Model for the regulation of *Arabidopsis thaliana* leaf margin development. Proceedings of the National Academy of Sciences, USA 108: 3424‐3429. 10.1073/pnas.1015162108 PMC3044365 21300866

[ajb216435-bib-0018] Blein, T. , A. Pulido , A. Vialette‐Guiraud , K. Nikovics , H. Morin , A. Hay , I. E. Johansen , et al. 2008. A conserved molecular framework for compound leaf development. Science 322: 1835‐1839. 19095941 10.1126/science.1166168

[ajb216435-bib-0019] Boer, H. J. d. , C. A. Price , F. Wagner‐Cremer , S. C. Dekker , P. J. Franks , and E. J. Veneklaas . 2016. Optimal allocation of leaf epidermal area for gas exchange. New Phytologist 210: 1219‐1228. 26991124 10.1111/nph.13929 PMC5069575

[ajb216435-bib-0020] Bonhomme, V. , S. Picq , C. Gaucherel , and J. Claude . 2014. Momocs: outline analysis using R. Journal of Statistical Software 56: 1–24.

[ajb216435-bib-0021] Borges, L. M. , V. C. Reis , and R. Izbicki . 2020. Schrödinger's phenotypes: Herbarium specimens show two‐dimensional images are both good and (not so) bad sources of morphological data. Methods in Ecology and Evolution 11: 1296‐1308.

[ajb216435-bib-0022] Bright, K. L. , and M. D. Rausher . 2008. Natural selection on a leaf‐shape polymorphism in the ivyleaf morning glory (*Ipomoea hederacea*). Evolution 62: 1978–1990. 18462212 10.1111/j.1558-5646.2008.00416.x

[ajb216435-bib-0023] Broadley, M. R. , and P. J. White . 2010. Eats roots and leaves. Can edible horticultural crops address dietary calcium, magnesium and potassium deficiencies? Proceedings of the Nutrition Society 69: 601‐612. 10.1017/S0029665110001588 20509990

[ajb216435-bib-0024] Budd, G. E. 2021. Morphospace. Current Biology 31: R1181–R1185. 34637728 10.1016/j.cub.2021.08.040

[ajb216435-bib-0025] Campitelli, B. E. , and J. R. Stinchcombe . 2013. Testing potential selective agents acting on leaf shape in *Ipomoea hederacea*: predictions based on an adaptive leaf shape cline. Ecology and Evolution 3: 2409–2423.

[ajb216435-bib-0026] Canales, C. , M. Barkoulas , C. Galinha , and M. Tsiantis . 2010. Weeds of change: *Cardamine hirsuta* as a new model system for studying dissected leaf development. Journal of Plant Research 123: 25‐33. 19821009 10.1007/s10265-009-0263-3

[ajb216435-bib-0027] Chen, Y. , F. Jabbour , A. Novikov , W. Wang , and S. Gerber . 2018. A study of floral shape variation in Delphinieae (Ranunculaceae) using geometric morphometrics on herbarium specimens. Botany Letters 165: 368‐376.

[ajb216435-bib-0028] Chitwood, D. H. , and W. C. Otoni . 2017. Morphometric analysis of *Passiflora* leaves: the relationship between landmarks of the vasculature and elliptical Fourier descriptors of the blade. GigaScience 6: giw008. 10.1093/gigascience/giw008 PMC5437945 28369351

[ajb216435-bib-0029] Chitwood, D. H. , A. Ranjan , C. C. Martinez , L. R. Headland , T. Thiem , R. Kumar , M. F. Covington , et al. 2014. A modern ampelography: a genetic basis for leaf shape and venation patterning in grape. Plant Physiology 164: 259‐272. 24285849 10.1104/pp.113.229708 PMC3875807

[ajb216435-bib-0030] Chitwood, D. H. , and N. R. Sinha . 2016. Evolutionary and environmental forces sculpting leaf development. Current Biology 26: R297‐R306. 27046820 10.1016/j.cub.2016.02.033

[ajb216435-bib-0031] Choi, B. , H. Jeong , and E. Kim . 2019. Phenotypic plasticity of *Capsella bursa‐pastoris* (Brassicaceae) and its effect on fitness in response to temperature and soil moisture. Plant Species Biology 34: 5‐10.

[ajb216435-bib-0032] Cochrane, A. , C. J. Yates , G. L. Hoyle , and A. B. Nicotra . 2015. Will among‐population variation in seed traits improve the chance of species persistence under climate change? Global Ecology and Biogeography 24: 12‐24.

[ajb216435-bib-0034] Consortium of Midwest Herbaria . 2024. Collections, Specimen search. Website https://midwestherbaria.org/portal/index.php [accessed August 2020–September 2021].

[ajb216435-bib-0036] Cornille, A. , A. Salcedo , D. Kryvokhyzha , S. Glémin , K. Holm , S. I. Wright , and M. Lascoux . 2016. Genomic signature of successful colonization of Eurasia by the allopolyploid shepherd's purse (*Capsella bursa‐pastoris*). Molecular Ecology 25: 616‐629. 26607306 10.1111/mec.13491

[ajb216435-bib-0037] Cornille, A. , M. Tiret , A. Salcedo , H. R. Huang , M. Orsucci , P. Milesi , D. Kryvokhyzha , et al. 2022. The relative role of plasticity and demographic history in *Capsella bursa‐pastoris*: a common garden experiment in Asia and Europe. AoB Plants 14: plac011. 35669442 10.1093/aobpla/plac011 PMC9162126

[ajb216435-bib-0038] Daru, B. H. , D. S. Park , R. B. Primack , C. G. Willis , D. S. Barrington , T. J. S. Whitfeld , T. G. Seidler , et al. 2018. Widespread sampling biases in herbaria revealed from large‐scale digitization. New Phytologist 217: 939‐955. 29083043 10.1111/nph.14855

[ajb216435-bib-0039] Davis, C. C. 2023. The herbarium of the future. Trends in Ecology Evolution 38: 412‐423. 36549958 10.1016/j.tree.2022.11.015

[ajb216435-bib-0040] de la Paz Pollicelli, M. , Y. L. Idaszkin , R. Gonzalez‐José , and F. M'arquez . 2018. Leaf shape variation as a potential biomarker of soil pollution. Ecotoxicology and Environmental Safety 164: 69‐74. 30098507 10.1016/j.ecoenv.2018.08.003

[ajb216435-bib-0041] de Villemereuil, P. , O. E. Gaggiotti , M. Mouterde , and I. Till‐Bottraud . 2016. Common garden experiments in the genomic era: new perspectives and opportunities. Heredity 116: 249‐254. 26486610 10.1038/hdy.2015.93 PMC4806574

[ajb216435-bib-0042] de Villemereuil, P. , O. E. Gaggiotti , and J. Goudet . 2022. Common garden experiments to study local adaptation need to account for population structure. Journal of Ecology 110: 1005‐1009.

[ajb216435-bib-0043] Dijksterhuis, G. B. , and J. C. Gower . 1991. The interpretation of generalized Procrustes analysis and allied methods. Food Quality and Preference 3: 67‐87.

[ajb216435-bib-0044] Ding, J. , E. A. Johnson , and Y. E. Martin . 2020. Optimization of leaf morphology in relation to leaf water status: a theory. Ecology and Evolution 10: 1510‐1525. 32076530 10.1002/ece3.6004 PMC7029057

[ajb216435-bib-0045] Dolph, G. E. , and D. L. Dilcher . 1980. Variation in leaf size with respect to climate in Costa Rica. Biotropica: 91‐99; 12.

[ajb216435-bib-0046] Du, J.‐X. , X.‐F. Wang , and G.‐J. Zhang . 2007. Leaf shape based plant species recognition. Applied Mathematics and Computation 185: 883‐893.

[ajb216435-bib-0047] Dujardin, J.‐P. , D. Kaba , P. Solano , M. Dupraz , K. D. McCoy , and N. Jaramillo‐O . 2014. Outline‐based morphometrics, an overlooked method in arthropod studies? Infection, Genetics and Evolution 28: 704‐714. 10.1016/j.meegid.2014.07.035 25111609

[ajb216435-bib-0048] Dunwiddie, P. W. 1985. Dichotomous key to conifer foliage in the Pacific Northwest. Northwest Science 59: 185–191.

[ajb216435-bib-0049] Chamberlain, S. , and D. Hocking . 2024. rnoaa: ‘NOAA’ weather data from R. R package version 1.4.0. Websites https://github.com/ropensci/rnoaa

[ajb216435-bib-0050] Enescu, C. M. 2017. A dichotomous determination key for autochthonous oak species from Romania. Journal of Horticulture, Forestry and Biotechnology 21: 58‐62.

[ajb216435-bib-0051] Feild, T. S. , T. L. Sage , C. Czerniak , and W. J. D. Iles . 2005. Hydathodal leaf teeth of *Chloranthus japonicus* (Chloranthaceae) prevent guttation‐induced flooding of the mesophyll. Plant Cell Environment 28: 1179‐1190.

[ajb216435-bib-0052] Felsenstein, J. 1973. Maximum‐likelihood estimation of evolutionary trees from continuous characters. American Journal of Human Genetics 25: 471. 4741844 PMC1762641

[ajb216435-bib-0054] Ferris, K. G. 2019. Endless forms most functional: uncovering the role of natural selection in the evolution of leaf shape. American Journal of Botany 106: 1532‐1535. 31797365 10.1002/ajb2.1398

[ajb216435-bib-0055] Ferris, K. G. , T. Rushton , A. B. Greenlee , K. Toll , B. K. Blackman , and J. H. Willis . 2015. Leaf shape evolution has a similar genetic architecture in three edaphic specialists within the *Mimulus guttatus* species complex. Annals of Botany 116: 213‐223. 26070644 10.1093/aob/mcv080 PMC4512191

[ajb216435-bib-0056] Ferris, K. G. , and J. H. Willis . 2018. Differential adaptation to a harsh granite outcrop habitat between sympatric *Mimulus* species. Evolution 72: 1225–1241. 29603731 10.1111/evo.13476

[ajb216435-bib-0058] Geeta, R. , L. M. D'avalos , A. Levy , L. Bohs , M. Lavin , K. Mummenhoff , N. Sinha , and M. F. Wojciechowski . 2012. Keeping it simple: flowering plants tend to retain, and revert to, simple leaves. New Phytologist 193: 481‐493. 22091556 10.1111/j.1469-8137.2011.03951.x

[ajb216435-bib-0059] Gleason, S. M. , C. J. Blackman , S. T. Gleason , K. A. McCulloh , T. W. Ocheltree , and M. Westoby . 2018. Vessel scaling in evergreen angiosperm leaves conforms with Murray's law and area‐filling assumptions: implications for plant size, leaf size and cold tolerance. New Phytologist 218: 1360‐1370. 29603233 10.1111/nph.15116

[ajb216435-bib-0060] Gower, J. C. 1975. Generalized Procrustes analysis. Psychometrika 40: 33‐51.

[ajb216435-bib-0061] Gregory‐Wodzicki, K. M. 2000. Relationships between leaf morphology and climate, Bolivia: implications for estimating paleoclimate from fossil floras. Paleobiology 26: 668‐688.

[ajb216435-bib-0062] Gupta, M. D. , and M. Tsiantis . 2018. Gene networks and the evolution of plant morphology. Current Opinion in Plant Biology 45: 82‐87. 29885565 10.1016/j.pbi.2018.05.011

[ajb216435-bib-0063] Gupta, S. , D. M. Rosenthal , J. R. Stinchcombe , and R. S. Baucom . 2020. The remarkable morphological diversity of leaf shape in sweet potato (*Ipomoea batatas*): the influence of genetics, environment, the influence of genetics, environment, and G×E. New Phytologist 225: 2183–2195. 31652341 10.1111/nph.16286

[ajb216435-bib-0064] Gutaker, R. M. , E. Reiter , A. Furtw¨angler , V. J. Schuenemann , and H. A. Burbano . 2017. Extraction of ultrashort DNA molecules from herbarium specimens. Biotechniques 62: 76‐79. 28193151 10.2144/000114517

[ajb216435-bib-0065] Hao, N. , J. Cao , C. Wang , Y. Zhu , Y. Du , and T. Wu . 2022. Understanding the molecular mechanism of leaf morphogenesis in vegetable crops conduces to breeding process. Frontiers in Plant Science 13: 971453. 36570936 10.3389/fpls.2022.971453 PMC9773389

[ajb216435-bib-0066] Harrison, E. L. , L. A. Cubas , J. E. Gray , and C. Hepworth . 2020. The influence of stomatal morphology and distribution on photosynthetic gas exchange. Plant Journal 101: 768‐779. 10.1111/tpj.14560 PMC7065165 31583771

[ajb216435-bib-0067] Heberling, J. M. , L. A. Prather , and S. J. Tonsor . 2019. The changing uses of herbarium data in an era of global change: an overview using automated content analysis. BioScience 69: 812‐822.

[ajb216435-bib-0068] Henn, J. J. , V. Buzzard , B. J. Enquist , A. H. Halbritter , K. Klanderud , B. S. Maitner , S. T. Michaletz , et al. 2018. Intraspecific trait variation and phenotypic plasticity mediate alpine plant species response to climate change. Frontiers in Plant Science 9: 1548. 30483276 10.3389/fpls.2018.01548 PMC6243391

[ajb216435-bib-0069] Huff, P. M. , P. Wilf , and E. J. Azumah . 2003. Digital future for paleoclimate estimation from fossil leaves? Preliminary results. Palaios 18: 266‐274.

[ajb216435-bib-0070] Hurka, H. , and B. Neuffer . 1997. Evolutionary processes in the genus Capsella (Brassicaceae). Plant Systematics and Evolution 206: 295‐316.

[ajb216435-bib-0071] Iannetta, P. P. M. , G. Begg , C. Hawes , M. Young , J. Russell , and G. R. Squire . 2007. Variation in *Capsella* (shepherd's purse): an example of intraspecific functional diversity. Physiologia Plantarum 129: 542‐554.

[ajb216435-bib-0072] Igual, L. , X. Perez‐Sala , S. Escalera , C. Angulo , and F. De la Torre . 2014. Continuous generalized Procrustes analysis. Pattern Recognition 47: 659‐671.

[ajb216435-bib-0073] Iia, P. , and N. G. Mikeshina . 2002. Principles and methods of geometric morphometrics. Zhurnal Obshchei Biologii 63: 473‐493. 12510587

[ajb216435-bib-0074] Jacques, F. M. B. , and Z. Zhou . 2010. Geometric morphometrics: a powerful tool for the study of shape evolution in Menispermaceae endocarps. Taxon 59: 881‐895.

[ajb216435-bib-0075] James, S. A. , P. S. Soltis , L. Belbin , A. D. Chapman , G. Nelson , D. L. Paul , and M. Collins . 2018. Herbarium data: global biodiversity and societal botanical needs for novel research. Applications in Plant Sciences 6: e1024. 29732255 10.1002/aps3.1024 PMC5851569

[ajb216435-bib-0076] John, G. P. , and C. J. Garnica‐Díaz . 2023. Embracing the complexity of leaf shape: a commentary on ‘Anatomical determinants of gas exchange and hydraulics vary with leaf shape in soybean’. Annals of Botany 131: i–iii. 10.1093/aob/mcad059 PMC10332391 37283295

[ajb216435-bib-0077] Karl, T. , and W. J. Koss . 1984. Report: regional and national monthly, seasonal, and annual temperature weighted by area, 1895–1983. Website https://repository.library.noaa.gov/view/noaa/10238

[ajb216435-bib-0078] Katifori, E. 2018. The transport network of a leaf. Comptes Rendus Physique 19: 244‐252.

[ajb216435-bib-0079] Kern, S. O. , M. J. Hovenden , and G. J. Jordan . 2004. The impacts of leaf shape and arrangement on light interception and potential photosynthesis in southern beech (*Nothofagus cunninghamii*). Functional Plant Biology 31: 471‐480. 32688919 10.1071/FP03211

[ajb216435-bib-0080] Key, S. , J. K. Ma , and P. M. Drake . 2008. Genetically modified plants and human health. Journal of the Royal Society of Medicine 101: 290‐298. 18515776 10.1258/jrsm.2008.070372 PMC2408621

[ajb216435-bib-0081] Kluyver, T. , B. Ragan‐Kelley , F. Pérez , B. Granger , M. Bussonnier , J. Frederic , K. Kelley , et al. 2016. Jupyter Notebooks – a publishing format for reproducible computational workflows. *In* F. Loizides and B. Schmidt [eds.], Positioning and power in academic publishing: players, agents and agendas, 87–90. IOS Press, Amsterdam, Netherlands.

[ajb216435-bib-0082] Koenig, D. , and D. Weigel . 2015. Beyond the thale: comparative genomics and genetics of *Arabidopsis* relatives. Nature Reviews Genetics 16: 285‐298. 10.1038/nrg3883 25854181

[ajb216435-bib-0083] Kusi, J. , and I. Karsai . 2020. Plastic leaf morphology in three species of *Quercus*: The more exposed leaves are smaller, more lobated and denser. Plant Species Biology 35: 24‐37.

[ajb216435-bib-0084] Lambeth, W. A. 1911. Trees, and how to know them: a manual with analytical and dichotomous keys of the principal forest trees of the South. BF Johnson Publishing, Richmond, VA, USA.

[ajb216435-bib-0085] Lang, P. L. M. , F. M. Willems , J. F. Scheepens , H. A. Burbano , and O. Bossdorf . 2019. Using herbaria to study global environmental change. New Phytologist 221: 110–122. 30160314 10.1111/nph.15401 PMC6585664

[ajb216435-bib-0086] Lavoie, C. 2013. Biological collections in an ever changing world: herbaria as tools for biogeographical and environmental studies. Perspectives in Plant Ecology, Evolution and Systematics 15: 68‐76.

[ajb216435-bib-0087] Lawing, A. M. , and P. D. Polly . 2010. Geometric morphometrics: recent applications to the study of evolution and development. Journal of Zoology 280: 1‐7.

[ajb216435-bib-0088] Li, M. , H. An , R. Angelovici , C. Bagaza , A. Batushansky , L. Clark , V. Coneva , et al. 2018. Topological data analysis as a morphometric method: using persistent homology to demarcate a leaf morphospace. Frontiers in Plant Science 9: 553. 29922307 10.3389/fpls.2018.00553 PMC5996898

[ajb216435-bib-0089] Li, Y. , D. Zou , N. Shrestha , X. Xu , Q. Wang , W. Jia , and Z. Wang . 2020. Spatiotemporal variation in leaf size and shape in response to climate. Journal of Plant Ecology 13: 87‐96.

[ajb216435-bib-0090] Lin, S. , K. J. Niklas , Y. Wan , D. Hölscher , C. Hui , Y. Ding , and P. Shi . 2020. Leaf shape influences the scaling of leaf dry mass vs. area: a test case using bamboos. Annals of Forest Science 77: 1‐15.

[ajb216435-bib-0091] Loiselle, B. A. , P. M. Jørgensen , T. Consiglio , I. Jiménez , J. G. Blake , L. G. Lohmann , and O. M. Montiel . 2008. Predicting species distributions from herbarium collections: does climate bias in collection sampling influence model outcomes? Journal of Biogeography 35: 105‐116.

[ajb216435-bib-0092] Love, J. M. , and K. G. Ferris . 2024. Local adaptation to an altitudinal gradient: the interplay between mean phenotypic trait variation and phenotypic plasticity in *Mimulus laciniatus* . Perspectives in Plant Ecology, Evolution and Systematics 63: 125795.

[ajb216435-bib-0093] Mazerolle, M. J. 2023. AICcmodavg: model selection and multimodel inference based on (Q)AIC(c). Website https://cran.r-project.org/package=AICcmodavg

[ajb216435-bib-0094] Meineke, E. K. , and B. H. Daru . 2021. Bias assessments to expand research harnessing biological collections. Trends in Ecology Evolution 36: 1071‐1082. 34489117 10.1016/j.tree.2021.08.003

[ajb216435-bib-0095] Menne, M. J. , I. Durre , R. S. Vose , B. E. Gleason , and T. G. Houston . 2012. An overview of the global historical climatology network‐daily database. Journal of Atmospheric and Oceanic Technology 29: 897‐910.

[ajb216435-bib-0096] Miller‐Rushing, A. J. , D. Primack , R. B. Primack , C. Imbres , and P. Del Tredici . 2004. Herbarium specimens as a novel tool for climate change research. Arnoldia 63: 26‐32.

[ajb216435-bib-0097] Mitteroecker, P. , and P. Gunz . 2009. Advances in geometric morphometrics. Evolutionary Biology 36: 235‐247.

[ajb216435-bib-0098] Moeller, M. , L.‐M. Gao , R. R. Mill , D.‐Z. Li , M. L. Hollingsworth , and M. Gibby . 2007. Morphometric analysis of the *Taxus wallichiana* complex (Taxaceae) based on herbarium material. Botanical Journal of the Linnean Society 155: 307‐335.

[ajb216435-bib-0099] Moerman, D. E. , and G. F. Estabrook . 2006. The botanist effect: counties with maximal species richness tend to be home to universities and botanists. Journal of Biogeography 33: 1969‐1974.

[ajb216435-bib-0100] Moloney, K. A. , C. Holzapfel , K. Tielbörger , F. Jeltsch , and F. M. Schurr . 2009. Rethinking the common garden in invasion research. Perspectives in Plant Ecology, Evolution and Systematics 11: 311‐320.

[ajb216435-bib-0101] Moran, E. V. , F. Hartig , and D. M. Bell . 2016. Intraspecific trait variation across scales: implications for understanding global change responses. Global Change Biology 22: 137‐150. 26061811 10.1111/gcb.13000

[ajb216435-bib-0102] Murdock, E. C. , J. B. Guice , and J. E. Toler . 1988. Identification of grass and morning glory species with simple dichotomous keys. Weed Technology 2: 198‐205.

[ajb216435-bib-0103] Neuffer, B. 1990. Ecotype differentiation in *Capsella* . Vegetation 89: 165‐171.

[ajb216435-bib-0104] Neuffer, B. , and M. Meyer‐Walf . 1996. Ecotypic variation in relation to man made habitats in *Capsella*: field and trampling area. Flora 191: 49‐57.

[ajb216435-bib-0105] Neuffer, B. , C. Wesse , I. Voss , and R. Scheibe . 2018. The role of ecotypic variation in driving worldwide colonization by a cosmopolitan plant. AoB Plants 10: ply005. 29479407 10.1093/aobpla/ply005 PMC5815058

[ajb216435-bib-0106] Nicotra, A. B. , M. J. Cosgrove , A. Cowling , C. D. Schlichting , and C. S. Jones . 2008. Leaf shape linked to photosynthetic rates and temperature optima in South African *Pelargonium* species. Oecologia 154: 625‐635. 17943318 10.1007/s00442-007-0865-1

[ajb216435-bib-0107] Orsucci, M. , P. Milesi , J. Hansen , J. Girodolle , S. Glémin , and M. Lascoux . 2020. Shift in ecological strategy helps marginal populations of shepherd's purse (*Capsella bursa‐pastoris*) to overcome a high genetic load. Proceedings of the Royal Society B 287: 20200463. 10.1098/rspb.2020.0463 PMC7287375 32429810

[ajb216435-bib-0108] Panchen, Z. A. , J. Doubt , H. M. Kharouba , and M. O. Johnston . 2019. Patterns and biases in an Arctic herbarium specimen collection: implications for phenological research. Applications in Plant Sciences 7: e01229. 30937221 10.1002/aps3.1229 PMC6426279

[ajb216435-bib-0109] Parins‐Fukuchi, C. 2018. Use of continuous traits can improve morphological phylogenetics. Systematic Biology 67: 328‐339. 28945906 10.1093/sysbio/syx072

[ajb216435-bib-0110] Park, D. S. , X. Feng , S. Akiyama , M. Ardiyani , N. Avendan˜o , Z. Barina , B. B¨artschi , et al. 2023. The colonial legacy of herbaria. Nature Human Behaviour 7: 1059‐1068. 10.1038/s41562-023-01616-7 37308536

[ajb216435-bib-0111] Parsons, K. J. , J. W. Cooper , and R. C. Albertson . 2009. Limits of principal components analysis for producing a common trait space: implications for inferring selection, contingency, and chance in evolution. PLoS One 4: e7957. 19956767 10.1371/journal.pone.0007957 PMC2776347

[ajb216435-bib-0112] Passioura, J. B. 1988. Water transport in and to roots. Annual Review of Plant Physiology and Plant Molecular Biology 39: 245‐265.

[ajb216435-bib-0114] Peppe, D. J. , D. L. Royer , B. Cariglino , S. Y. Oliver , S. Newman , E. Leight , G. Enikolopov , et al. 2011. Sensitivity of leaf size and shape to climate: global patterns and paleoclimatic applications. New Phytologist 190: 724‐739. 21294735 10.1111/j.1469-8137.2010.03615.x

[ajb216435-bib-0053] Perez, F. , and B. E. Granger . 2007. IPython: a system for interactive scientific computing. Computing in Science and Engineering 9: 21‐29.

[ajb216435-bib-0115] Peterson, T. C. , R. Vose , R. Schmoyer , and V. Razuväev . 1998. Global Historical Climatology Network (GHCN) quality control of monthly temperature data. International Journal of Climatology 18: 1169‐1179.

[ajb216435-bib-0116] Pettigrew, W. T. , and T. J. Gerik . 2007. Cotton leaf photosynthesis and carbon metabolism. Advances in Agronomy 94: 209–236.

[ajb216435-bib-0117] Pettigrew, W. T. , J. J. Heitholt , and K. C. Vaughn . 1993. Gas exchange differences and comparative anatomy among cotton leaf‐type isolines. Crop Science 33: 1295‐1299.

[ajb216435-bib-0118] Polly, P. D. , and G. J. Motz . 2016. Patterns and processes in morphospace: geometric morphometrics of three‐dimensional objects. Paleontological Society Papers 22: 71‐99.

[ajb216435-bib-0119] Pritchard, S. G. , H. H. Rogers , S. A. Prior , and C. M. Peterson . 1999. Elevated CO_2_ and plant structure: a review. Global Change Biology 5: 807‐837.

[ajb216435-bib-0120] Quinteros, P. A. , C. I. Goloboff , and A. S. Mattoni . 2006. Continuous characters analyzed as such. Cladistics 22: 589‐601. 34892898 10.1111/j.1096-0031.2006.00122.x

[ajb216435-bib-0121] R Core Team . 2021. R: a language and environment for statistical computing. R Foundation for Statistical Computing, Vienna, Austria. Website https://www.R-project.org/

[ajb216435-bib-0122] R Studio Team . 2020. RStudio: integrated development environment for R. RStudio, PBC, Boston, MA, USA. Website http://www.rstudio.com/

[ajb216435-bib-0123] Richards, T. J. , D. Ortiz‐Barrientos , and K. McGuigan . 2019. Natural selection drives leaf divergence in experimental populations of *Senecio lautus* under natural conditions. Ecology and Evolution 9: 6959‐6967. 31380026 10.1002/ece3.5263 PMC6662321

[ajb216435-bib-0124] Rivero‐Lynch, A. P. , V. K. Brown , and J. H. Lawton . 1997. The impact of leaf shape on the feeding preference of insect herbivores: experimental and field studies with *Capsella* and *Phyllotreta* . Philosophical Transactions of the Royal Society, B, Biological Sciences 351: 1671‐1677.

[ajb216435-bib-0125] Royer, D. L. 2012. Leaf shape responds to temperature but not CO_2_ in *Acer rubrum* . PLoS One 7: e49559. 23152921 10.1371/journal.pone.0049559 PMC3495865

[ajb216435-bib-0126] Royer, D. L. , J. C. McElwain , J. M. Adams , and P. Wilf . 2008. Sensitivity of leaf size and shape to climate within *Acer rubrum* and *Quercus kelloggii* . New Phytologist 179: 808‐817. 18507771 10.1111/j.1469-8137.2008.02496.x

[ajb216435-bib-0127] Royer, D. L. , L. A. Meyerson , K. M. Robertson , and J. M. Adams . 2009. Phenotypic plasticity of leaf shape along a temperature gradient in *Acer rubrum* . PLoS One 4: e7653. 19893620 10.1371/journal.pone.0007653 PMC2764093

[ajb216435-bib-0128] Runions, A. , M. Tsiantis , and P. Prusinkiewicz . 2017. A common developmental program can produce diverse leaf shapes. New Phytologist 216: 401‐418. 28248421 10.1111/nph.14449 PMC5638099

[ajb216435-bib-0129] Sakurai, G. , and S. J. Miklavcic . 2021. On the efficacy of water transport in leaves. A coupled xylem‐phloem model of water and solute transport. Frontiers in Plant Science 12: 615457. 33613602 10.3389/fpls.2021.615457 PMC7889512

[ajb216435-bib-0130] Sang‐Hun, O. H. 2022. Morphometric analysis of the *Daphne kiusiana* complex (Thymelaeaceae) using digitized herbarium specimens. Korean Journal of Plant Taxonomy 52: 144‐155.

[ajb216435-bib-0131] Schindelin, J. , I. Arganda‐Carreras , E. Frise , V. Kaynig , M. Longair , T. Pietzsch , S. Preibisch , et al. 2012. Fiji: an open‐source platform for biological‐image analysis. Nature Methods 9: 676‐682. 22743772 10.1038/nmeth.2019 PMC3855844

[ajb216435-bib-0132] Schrader, J. , P. Shi , D. L. Royer , D. J. Peppe , R. V. Gallagher , Y. Li , R. Wang , and I. J. Wright . 2021. Leaf size estimation based on leaf length, width and shape. Annals of Botany 128: 395‐406. 34157097 10.1093/aob/mcab078 PMC8414912

[ajb216435-bib-0133] Shi, P. , M. Liu , X. Yu , J. Gielis , and D. A. Ratkowsky . 2019. Proportional relationship between leaf area and the product of leaf length and width of four types of special leaf shapes. Forests 10: 178.

[ajb216435-bib-0134] Shull, G. H. 1909. Bursa bursa‐pastoris and Bursa heegeri biotypes and hybrids. Carnegie Institution of Washington, Washington, D.C., USA.

[ajb216435-bib-0135] Sicard, A. , A. Thamm , C. Marona , Y. W. Lee , V. Wahl , J. R. Stinchcombe , S. I. Wright , et al. 2014. Repeated evolutionary changes of leaf morphology caused by mutations to a homeobox gene. Current Biology 24: 1880‐1886. 25127212 10.1016/j.cub.2014.06.061

[ajb216435-bib-0137] Smith, W. K. , T. C. Vogelmann , E. H. DeLucia , D. T. Bell , and K. A. Shepherd . 1997. Leaf form and photosynthesis. BioScience 47: 785‐793.

[ajb216435-bib-0138] Sparks, A. H. , T. Hengl , and A. Nelson . 2017. GSODR: global summary daily weather data in R. Journal of Open Source Software 2: 177.

[ajb216435-bib-0139] Streubel, S. , M. A. Fritz , M. Teltow , C. Kappel , and A. Sicard . 2018. Successive duplication‐divergence mechanisms at the RCO locus contributed to leaf shape diversity in the Brassicaceae. Development 145: dev164301. 29691226 10.1242/dev.164301

[ajb216435-bib-0140] Tamang, B. G. , Y. Zhang , M. A. Zambrano , and E. A. Ainsworth . 2023. Anatomical determinants of gas exchange and hydraulics vary with leaf shape in soybean. Annals of Botany 131: 909‐920. 36111999 10.1093/aob/mcac118 PMC10332398

[ajb216435-bib-0141] Thyagharajan, K. K. , and I. Kiruba Raji . 2019. A review of visual descriptors and classification techniques used in leaf species identification. Archives of Computational Methods in Engineering 26: 933‐960.

[ajb216435-bib-0144] Tsukaya, H. 2018. A consideration of leaf shape evolution in the context of the primary function of the leaf as a photosynthetic organ. In W. W. Adams III and I. Terashima [eds.], The leaf: a platform for performing photosynthesis, 1–26. Springer, Cham, Switzerland.

[ajb216435-bib-0145] Valenzuela, A. J. F. , B. S. Santos , J. P. Quilang , and E. P. Cao . 2011. Comparative use of meristic and geomorphometric analyses of vegetative and floral characters in studying intraspecific variation in *Portulaca grandiflora* (Hook). Science Diliman 23: 41‐53.

[ajb216435-bib-0146] Vergeer, P. , and W. E. Kunin . 2011. Life history variation in *Arabidopsis lyrata* across its range: effects of climate, population size and herbivory. Oikos 120: 979‐990.

[ajb216435-bib-0148] Walter, G. M. , J. Clark , D. Terranova , S. Cozzolino , A. Cristaudo , S. J. Hiscock , and J. Bridle . 2023. Hidden genetic variation in plasticity provides the potential for rapid adaptation to novel environments. New Phytologist 239: 374–387. 36651081 10.1111/nph.18744

[ajb216435-bib-0149] Webster, M. , and D. H. Sheets . 2010. A practical introduction to landmark‐based geometric morphometrics. Paleontological Society Papers 16: 163‐188.

[ajb216435-bib-0150] Wesse, C. , E. Welk , H. Hurka , and B. Neuffer . 2021. Geographical pattern of genetic diversity in Capsella bursa‐pastoris (Brassicaceae)—A global perspective. Ecology and Evolution 11: 199‐213. 33437423 10.1002/ece3.7010 PMC7790636

[ajb216435-bib-0151] Wiemann, M. C. , S. R. Manchester , D. L. Dilcher , L. F. Hinojosa , and E. A. Wheeler . 1998. Estimation of temperature and precipitation from morphological characters of dicotyledonous leaves. American Journal of Botany 85: 1796‐1802. 21680340

[ajb216435-bib-0152] Williams, J. , and K. D. Pearson . 2019. Examining collection biases across different taxonomic groups: understanding how biases can compare across herbarium datasets. American Journal of Undergraduate Research 15: 47‐53.

[ajb216435-bib-0153] Willis, C. G. , E. R. Ellwood , R. B. Primack , C. C. Davis , K. D. Pearson , A. S. Gallinat , J. M. Yost , et al. 2017. Old plants, new tricks: phenological research using herbarium specimens. Trends in Ecology Evolution 32: 531‐546. 28465044 10.1016/j.tree.2017.03.015

[ajb216435-bib-0154] Wilson Brown, M. K. , and E. B. Josephs . 2023. Evaluating niche changes during invasion with seasonal models in *Capsella bursa‐pastoris* . American Journal of Botany 110: e16140. 10.1002/ajb2.16140 PMC10088061 36758170

[ajb216435-bib-0155] Winn, A. A. 1999. The functional significance and fitness consequences of heterophylly. International Journal of Plant Sciences 160: S113‐S121. 10572026 10.1086/314222

[ajb216435-bib-0156] Wu, Y. , and R. I. Colautti . 2022. Evidence for continent‐wide convergent evolution and stasis throughout 150y of a biological invasion. Proceedings of the National Academy of Sciences, USA 119: e2107584119. 10.1073/pnas.2107584119 PMC9170017 35476511

[ajb216435-bib-0158] Zeileis, A. , and G. Grothendieck . 2005. zoo: S3 Infrastructure for regular and irregular time series. Journal of Statistical Software 14: 1–27. 10.18637/jss.v014.i06

[ajb216435-bib-0159] Zhang, X. , W. Zhao , H. Luo , L. Chen , J. Peng , and J. Fan . 2019. Plant recognition via leaf shape and margin features. Multimedia Tools and Applications 78: 27463‐27489.

[ajb216435-bib-0160] Zwieniecki, M. A. , K. C. Boyce , and M. N. Holbrook . 2004. Functional design space of single‐veined leaves: role of tissue hydraulic properties in constraining leaf size and shape. Annals of Botany 94: 507‐513. 15319225 10.1093/aob/mch173 PMC4242227

