# Supplementary material for: Herbarium specimens reveal links between leaf shape of Capsella bursa‐pastoris and climate
Source: Am J Bot. 2024 Nov 6;111(11):e16435. doi: 10.1002/ajb2.16435 (PMC11584044; doi:10.1002/ajb2.16435)
Supplement: Supplementary file 2 — Appendix S2. Circularity and aspect ratio cluster within the morphospace PCA. [file AJB2-111-e16435-s005.pdf]

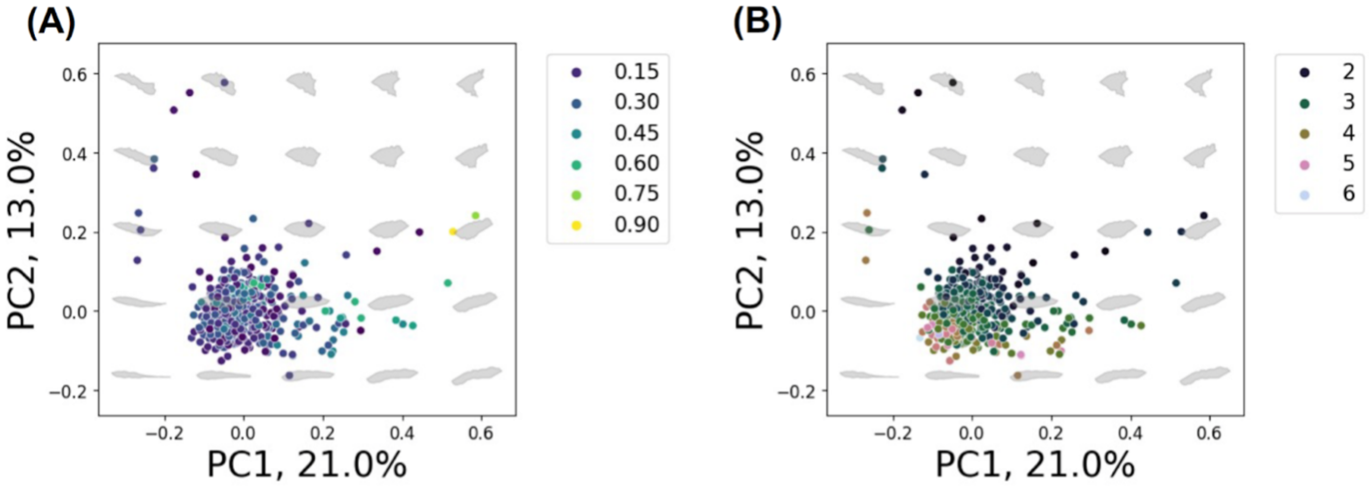

Appendix S2 - **Circularity and aspect ratio cluster within the morphospace PCA**. A,B. Morphospace PCA of circularity (A) and aspect ratio (B). Circularity colors range continuously from blue (lowest circularity, most lobed) to yellow (most circular, least lobed). Aspect ratio colors range continuously from purple (lowest, wider and shorter shapes) to light blue (highest, thinnest and longest shapes).
