# Supplementary figures and images for: Herbarium specimens reveal links between leaf shape of Capsella bursa‐pastoris and climate
Source: Am J Bot. 2024 Nov 6;111(11):e16435. doi: 10.1002/ajb2.16435 (PMC11584044; doi:10.1002/ajb2.16435)

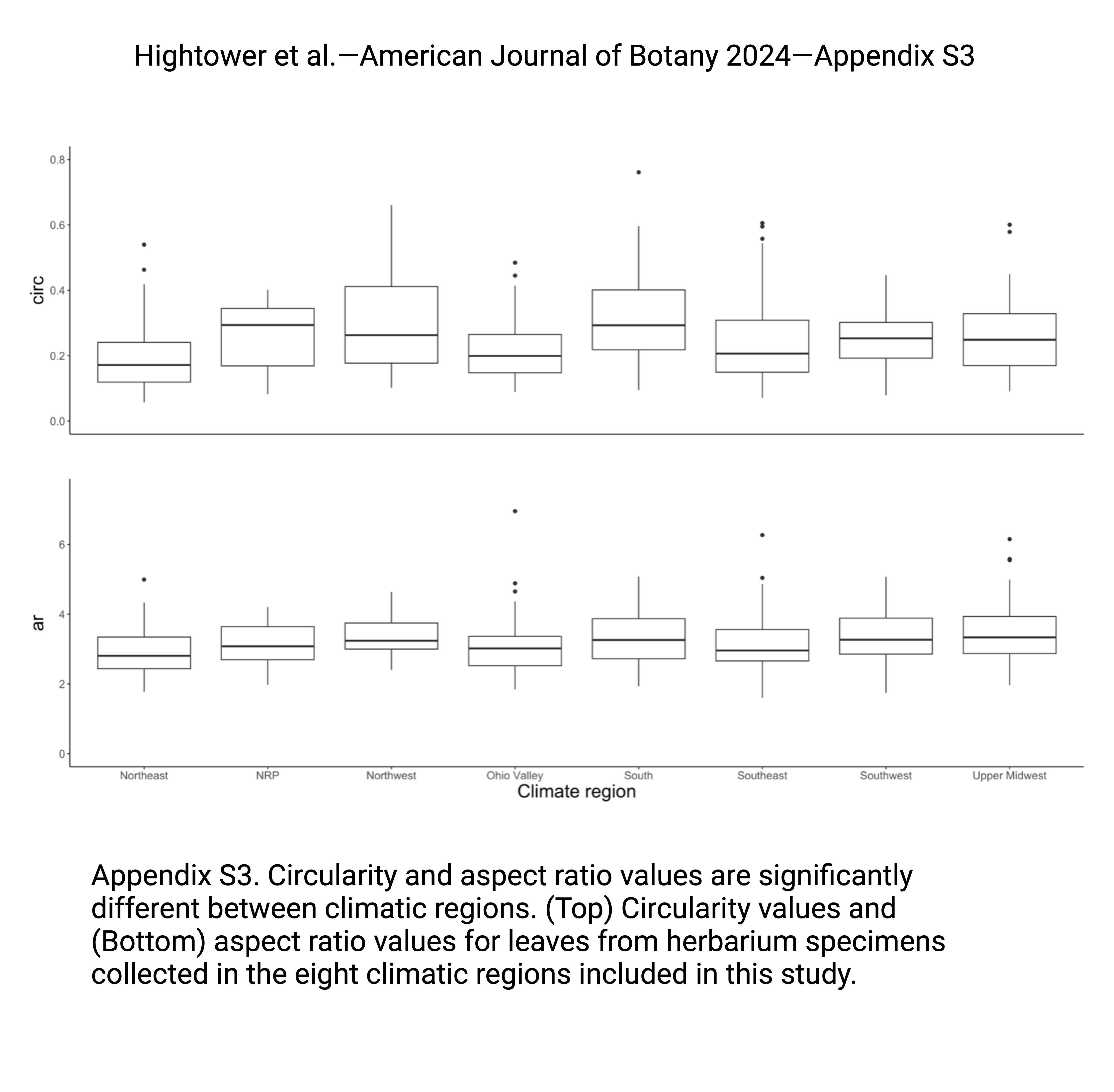

Supplement: Supplementary file 3 — Appendix S3. Circularity and aspect ratio values are significantly different between climatic regions. [file AJB2-111-e16435-s006.png]

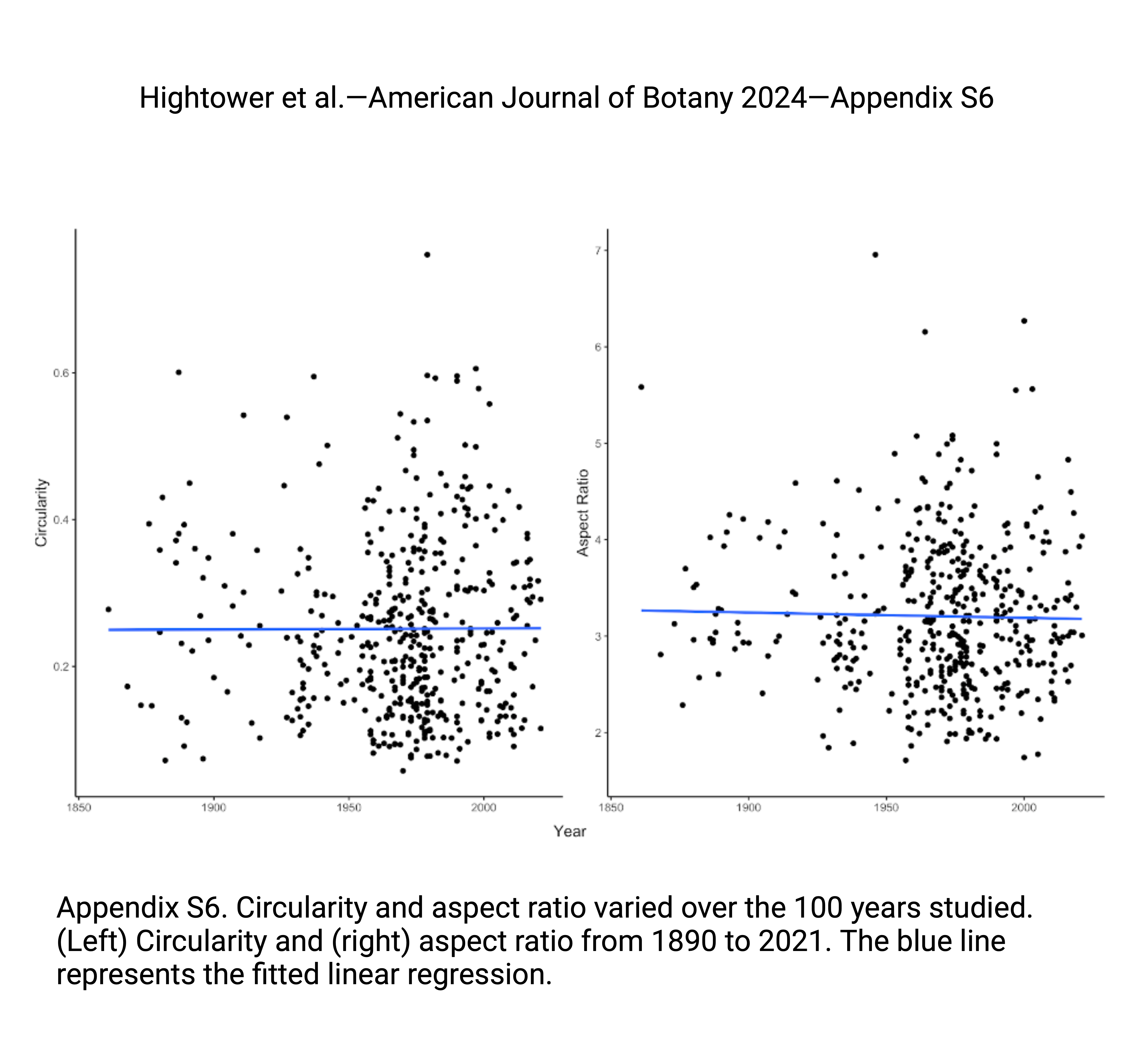

Supplement: Supplementary file 6 — Appendix S6. Circularity and aspect ratio varied over the 100 years studied. [file AJB2-111-e16435-s002.png]
