# Supplementary material for: Herbarium specimens reveal links between leaf shape of Capsella bursa‐pastoris and climate
Source: Am J Bot. 2024 Nov 6;111(11):e16435. doi: 10.1002/ajb2.16435 (PMC11584044; doi:10.1002/ajb2.16435)
Supplement: Supplementary file 4 — Appendix S4. Circularity is strongly associated with average growing season temperature and by climate region. [file AJB2-111-e16435-s003.pdf]

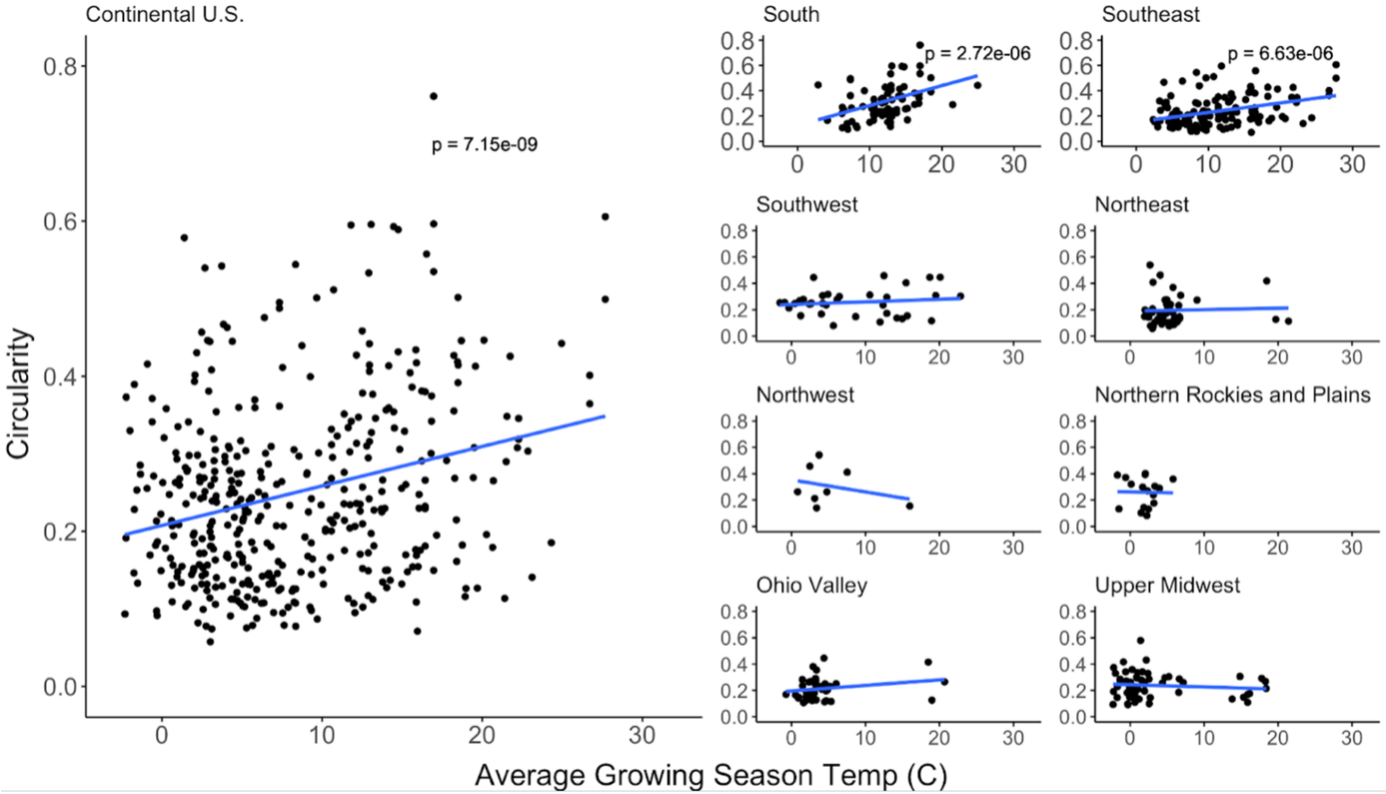

Appendix S4 - **Circularity is strongly associated with average growing season temperature and by climate region.** Blue line represents fitted linear regression. P values for linear regression are provided for the continental U.S. and regions that are significantly associated with circularity over the average growing season temperature.
