# Supplementary material for: Herbarium specimens reveal links between leaf shape of Capsella bursa‐pastoris and climate
Source: Am J Bot. 2024 Nov 6;111(11):e16435. doi: 10.1002/ajb2.16435 (PMC11584044; doi:10.1002/ajb2.16435)
Supplement: Supplementary file 5 — Appendix S5. Sample sizes of individuals collected from each climate region. [file AJB2-111-e16435-s007.pdf]

Climate Region	Number of Individuals
Northeast	51
Northern Rockies and Plains	20
Northwest	16
Ohio Valley	57
South	78
Southeast	152
Southwest	40
Upper Midwest	82

Appendix S5 - Sample sizes of individuals collected from each climate region.
